# The protein-water H-bond network for bacterial response regulator REC domain activation

**DOI:** 10.1016/j.yjsbx.2026.100164

**Published:** 2026-09-19

**Authors:** Maham Hamid, Ishrat Jabeen, Safee Ullah Chaudhary, Shahid Khan

**Affiliations:** a Biomedical Informatics and Engineering Research Laboratory, Department of Life Sciences, Syed Babar Ali School of Science and Engineering, Lahore University of Management Sciences, Lahore 54792, Pakistan; b National University of Sciences and Technology (NUST), 44000 Islamabad, Pakistan; c Advanced Light Source, Lawrence Berkeley National Laboratory, Berkeley, CA 94720, USA; d Syed Babar Ali School of Science and Engineering, Lahore University of Management Sciences, Lahore 54792, Pakistan

**Keywords:** Protein allostery, Energy frustration, Switch residues, Phosphorylation site solvation

## Abstract

The bacterial response regulator (RR) superfamily is activated by aspartyl phosphorylation of the receiver (REC) domain for diverse functions. Crystal structures of the enteric CheY chemotaxis RRs (CheY_es_) have revealed water-residue hydrogen bonds (H-bonds) essential for activation. Here, we compared the H-bond networks, computed from MD simulations, of the basal and phosphorylated conformational landscapes in silico. Representative REC domains were selected by filtering the sequence and structure RR databases based on sequence information and fold variability. Residues bonded with water included the aspartate triad for divalent metal ion coordination and phosphoryl transfer, plus K109. The water-residue H-bond propensity of these residues was balanced by the impaired stability of adjacent residue contacts maintained by evolution. Upon phosphorylation, the CheY_es_ aspartyl phosphate (D57-PO_3_^−^) became the major node, connected via the conserved switch residues K109, T87 to the distant target binding surface with H-bond and associated long-range couplings measured by mutual information. The communication was mediated by transient H-bonded chains that included internal water in the hydrophobic core. Study of two other RR representatives indicates that an increased D57-PO_3_^−^ node strength in the REC protein-water H-bond network upon phosphorylation is a generic feature of RR allostery. There are subtle differences in the connectivity between D57 and an aromatic residue (CheY_es_ Y106 or equivalent), diagnostic of interfacial dynamics. These differences, influenced by the water contribution, may reflect function-related spatiotemporal constraints useful for the design of RR subfamily-specific, allosteric inhibitors.

## Highlights

• REC database representatives extracted from sequence, fold, and functional similarity.
• Evolution of the water-rich REC phosphorylation sites is maximally frustrated.
• The aspartyl phosphate orchestrates phosphorylated REC H-bond networks.
• Molecular dynamics detects internal water and multiple residue H-bond states.
• Y-T coupling is not conserved across three representative RRs.

## Graphical abstract

**Figure:**
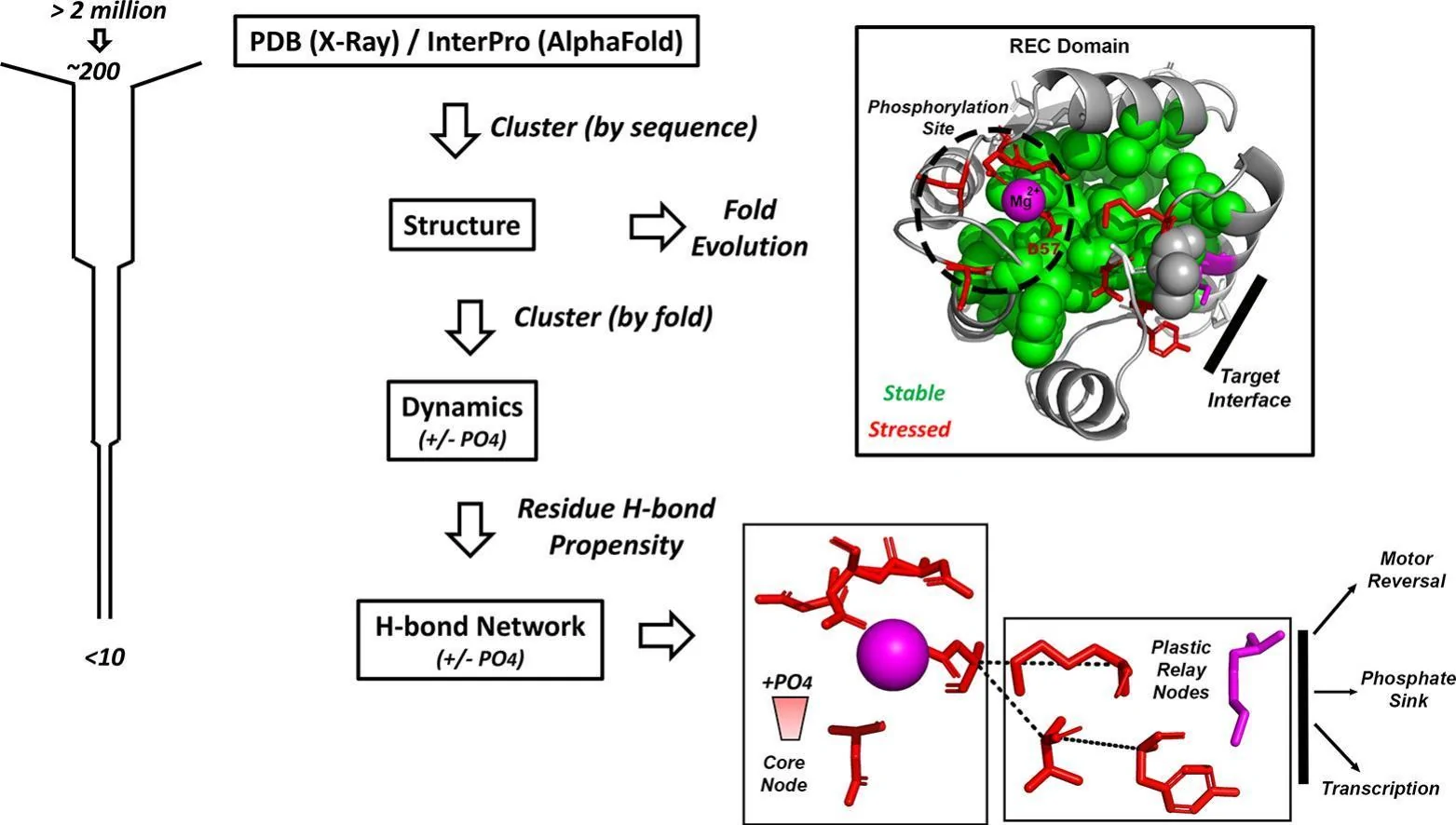
Representation, construction and properties of the REC H-bond network.

## 1 Introduction

Water is an integral component of protein H-bonded networks. Bound, or structural, waters, long-recognized as important for protein folding (Papoian et al., 2004), are increasingly appreciated as critical for allosteric communication (Bondar, 2022; Bondar and DeCoursey, 2024). Numerous tools have been developed for the computational analysis of bound waters (Samways et al., 2021; Spyrakis et al., 2017). Water-mediated H-bonds were visualized in the collagen triple helix over two decades ago (Bella et al., 1994). Since then, water has been shown to stabilize prions (De Simone et al., 2006; De Simone et al., 2005), while flickering water wires mediate ion channel selectivity (Kratochvil et al., 2023; Paulino et al., 2020).

Here, we apply two recent tools, Frustraevo (Freiberger et al., 2023) and Bridge2 (Siemers and Bondar, 2021) to characterize the role of bound waters on stability and allostery of the bacterial response regulator (RR) superfamily of sensory proteins. Frustraevo evaluates protein stability from structural models of superfamily members. Water-mediated contacts, computed with the associated memory, water-mediated structure and energy model (AWSEM) (Davtyan et al., 2012)) are important determinants. The Frustraevo predictions correlate with experimental measurements of protein stability (Faure et al., 2022). Bridge2 reports protein-water, as well as protein-protein hydrogen bond (H-bond) temporal networks from molecular dynamics (MD) trajectories of atomic protein structures. It has given important insights into H-bonded water wires in receptors (Bertalan et al., 2020; Lesnik et al., 2023), transporters (Lazaratos et al., 2022) and porins (Drewniak et al., 2024).

The RR superfamily mediates diverse responses that dictate how bacteria interact with their environment (Gao et al., 2019). Most members bind DNA and, to a lesser extent, protein and RNA for functions that include gene regulation, metabolism, phosphate regulation, osmosensing, and chemotaxis (Gao et al., 2007). In the years since the first structures of the canonical enteric *Escherichia Coli* (*E. coli*) and *Salmonella enterica* serovar Typhimurium (*S. typhimurium*) CheY chemotaxis proteins were published (Stock et al., 1989; Volz and Matsumura, 1991); over 2 million sequences and 100 near-atomic (< 2 Å resolution) crystal structures of superfamily members have been deposited, making it the ninth most abundant superfamily in protein databases (Kennedy et al., 2022).

RR signal transduction is based on phosphorylation of a conserved aspartyl residue (D57), as first demonstrated, and best understood, for the enteric *E. coli / S. typhimurium* CheY chemotaxis proteins (>98% sequence identity) (Bourret et al., 1990; Lukat et al., 1991), henceforth jointly referred to as “CheY_es_*”.* Activation by dephosphorylation can also occur (Branscum et al., 2019). In this study, CheY_es_ numbering, based on multiple sequence alignments (MSAs) will apply to other RR REC residue positions, unless otherwise specified. Phosphoryl group transfer from a cognate kinase to the aspartate (CheY_es_ D57) carboxyl forms a high-energy acyl phosphate (aspartyl phosphate (Cavanagh et al., 1994)) that triggers changes at a distant target binding surface (Cho et al., 2000; Lee et al., 2001a; Zhu et al., 1997a). The fluorescence quenching of W58, adjacent to D57, is a classic indicator of activation (Lukat et al., 1992), with W58 motions refined by later experiments (McDonald et al., 2012; Wheatley et al., 2020). Metal ions are an essential cofactor (Gao et al., 2019; Stock et al., 1993). RRs are likely to exist in their Mg^2+^ bound form in vivo since Mg^2+^ is the most abundant divalent cation in prokaryotes (Franken et al., 2022); but Mn^2+^ and other divalent ions have been used to obtain crystal structures. There are five conserved residues (D12, D13, D57, K109, T87) in the RR superfamily (Gao et al., 2019). D57 phosphorylation requires Mg^2+^ coordination with D12, D13 (Stock et al., 1993), forming the “phosphorylation site”. K109 forms an activation-dependent salt-bridge with D57. Coordinated movement of T87 with Y106 at the target binding surface, termed Y-T coupling, mediates communication between the phosphorylation site and this surface (Dyer et al., 2004). T87 residue substitutions uncouple phosphorylation from chemotaxis (Ganguli et al., 1995; Jiang et al., 1997). Internal Y106 sidechain rotation correlates with chemotactic response (Zhu et al., 1997b) in most, but not all cases. The Y106 is not conserved, and both internal and external orientations are seen in unphosphorylated, particularly DNA-binding, RRs (Gao et al., 2019).

The acyl phosphate is labile, so beryllium fluoride (BeF_3_^−^), a phosphoryl analog (Petsko, 2000) is commonly used to mimic the activated state (Lee et al., 2001a). BeF_3_^−^ elicits further changes in residue and water interactions around D57 (Cho et al., 2000; Lee et al., 2001a) and triggers changes at a distant binding surface (Cho et al., 2000; Lee et al., 2001a; Zhu et al., 1997a) with or without the bound target, as determined for the motor protein FliM (Lee et al., 2001b). Subsequent work showed that the CheA kinase and CheZ binding surfaces overlap with the FliM interface (McEvoy et al., 1999). Other RRs have important structural and biochemical differences from CheY_es_. Whereas CheY_es_ RRs are comprised of the receiver (REC) domain alone, many RRs have other domains concatenated with single or multiple REC domains. The aspartyl phosphorylation triggers different outcomes. For example, it facilitates homodimerization in the transcriptional regulator OmpR/PhoB family to increase DNA affinity (Toro-Roman et al., 2005). The dimerization surface overlaps with the binding interface for the CheY_es_ chemotaxis proteins.

A library of CheY_es_ crystal structures with residue substitutions has linked conformation to chemotactic function (Table S2 (Wheatley et al., 2020)). Dynamic H-bonds are central to CheY_es_, allosteric coupling ((Fersht et al., 1985; Gao et al., 2019), and references therein), as for many protein families (Fersht et al., 1985). The evidence ((Schuster et al., 2001; Wheatley et al., 2020) and references therein), indicates that CheY_es_ employs conformational selection of a heterogeneous basal population instead of a concerted transition for mechanistic activation (Gao et al., 2019). Acetylation of the conserved K109, and K91, enhances motor CW rotation analogous to phosphorylation (Fraiberg et al., 2015). Database-guided mutagenesis (Kennedy et al., 2022) has shown RR phosphorylation/dephosphorylation kinetics, while rapid for CheY_es_ (Foster et al., 2021; Straughn et al., 2020), consistent with the sub-second chemotactic excitation and adaptation kinetics (Fukuoka et al., 2014; Fukuoka et al., 2024; Khan et al., 1995; Sagawa et al., 2014). vary by 5 or more orders of magnitude between family members (Immormino et al., 2016; Page et al., 2016). Our X-ray foot printing data on sidechain solvent accessibility, supplemented with MD-derived, C^a^-based community networks (Wheatley et al., 2020) reported internalization of aromatic sidechains and loop immobilization increased CheY_es_ FliM affinity, consistent with structural data on CheY_es_ and homologs (Ahn et al., 2013). Activation, as used in this study, is functional activation; different from a concerted switching mechanism (McDonald et al., 2012; Wang et al., 2020).

These seminal insights into the allosteric communication between the phosphorylation site and the target surface are nevertheless incomplete. Our (Wheatley et al., 2020) and other MD studies on CheY_es_ (Foster and West, 2017; Foster et al., 2021) and references therein) or other RRs characterized the C^α^ conformational landscape but did not consider H-bond dynamics. The solvation of the phosphorylation site was measured in native CheY_es_ and a D13KY106W double-mutant phosphomimic (Hamid et al., 2022) but the differences were comprised by the D13K substitution. Molecular CheY_es_ structures have been correlated with behavioral phenotypes, but there is a sharp decrease in such knowledge for other RRs. The unambiguous determination of H-bonds and water molecule positions in experimental structures is beset with challenges (Prisant et al., 2020; Schiebel et al., 2018), despite notable successes (Giannetti et al., 2025; Tani and Fujiyoshi, 2014); and there is no knowledge of water dynamics in RRs to alleviate these challenges.

Here, we present simulations of the basal (Mg^2+^) and phosphorylated (Mg^2+^/D57_PO3_^−^) CheY conformational landscapes in silico (Hamid et al., 2026) with Frustraevo and Bridge2. We simulated CheY_es_ basal (2CHE.pdb (Stock et al., 1993)) and phosphorylated configurations created from BeF_3_^−^-modified 1FQW.pdb (Lee et al., 2001a), henceforth distinguished by the subscript PO3- (i.e. 1FQW_PO3-_). Residue H-bond propensities for these and selected representatives of the RR superfamily were correlated with their energy frustration profiles. Protein-water H-bond networks between the phosphorylation site and the common target binding interface, and the associated long-range dynamic couplings, were then constructed for CheY_es_ and representatives of the two major clusters. The networks extracted common features as well as subtle differences in the modulation of fold stability, the phosphorylation site, and the allosteric network linking it to the target binding surface.

## 2 Results and discussion

### 2.1 The conformational landscape of the REC domain

#### 2.1.1 Dynamics of basal and phosphorylated CheY_es_

We confirmed that our in silico phosphoryl (PO_3_^−^) modified structure (1FQW_PO3-_) reported the changed angles and bond distances from the basal configuration visualized in BeF_3_^−^-modified CheY_es_ (1FQW), a mimic of the phosphorylated active state (Lee et al., 2001a) (Fig. 1A). See also (Foster and West, 2017). The Mn^2+^ in the 1FQW crystal structure was replaced with Mg^2+^ in addition to removal of the BeF_3_^−^ to obtain 1FQW_PO3-_. The Y106 internal rotation coupled to T87 reorientation, first seen in BeF_3_^−−^-modified CheY_es_, has been a diagnostic of CheY_es_ activation. Upon in silico phosphorylation, 1FQW_PO3-_ Y106 and T87 had an inward orientation relative to the basal form (2CHE); D57 bonded with K109, T87 and N59, while Y106 bonded with E89, all as observed in the BeF_3_^−−^-modified configuration.

**Fig. 1:**
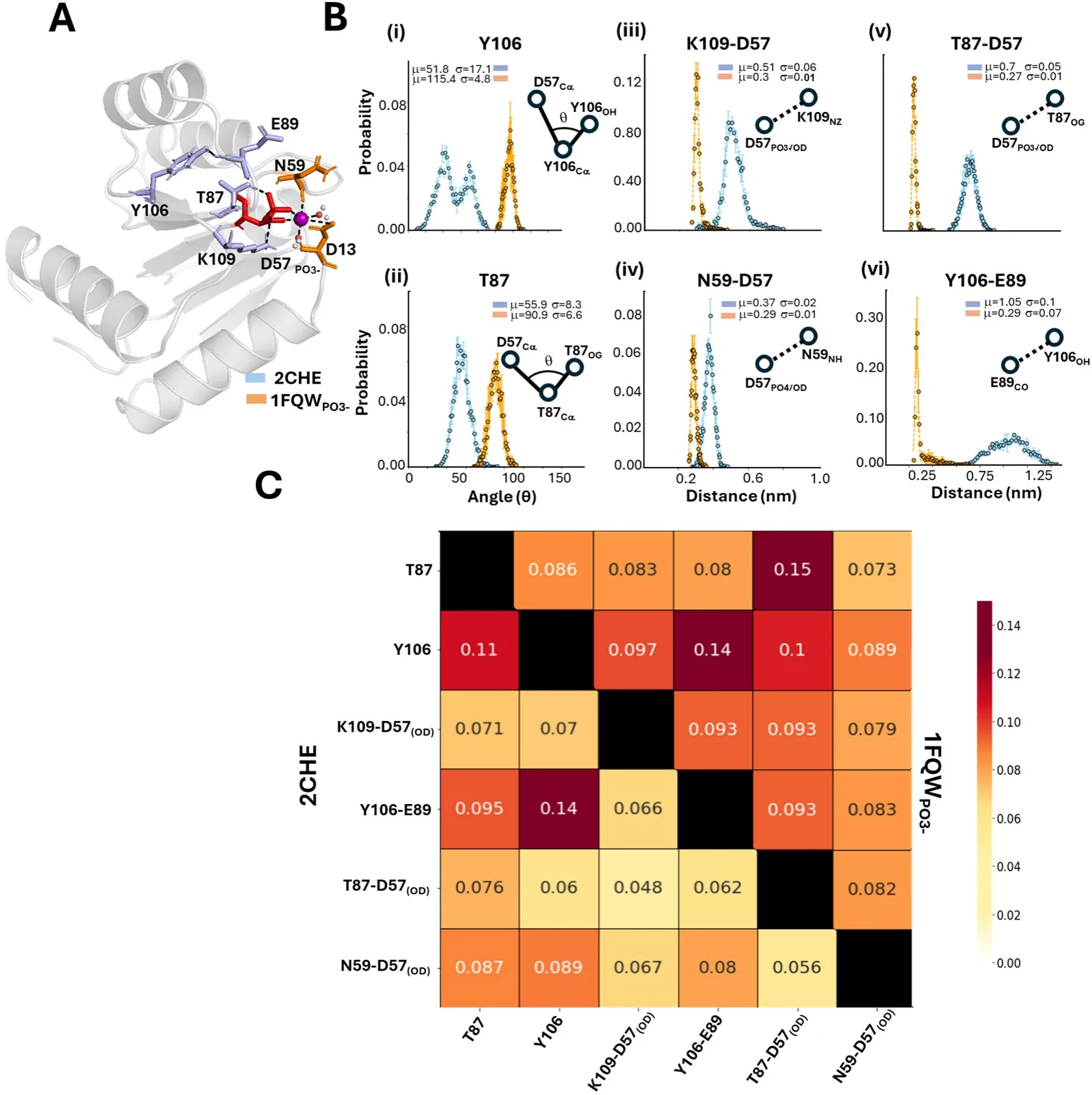
MD simulations of Mg^2+^ bound CheY_es_ structures with and without D57_PO3-_. A. Schematic of the phosphorylation site. The bonds formed by the Mg^2+^ ion (magenta sphere) and the -PO3^−^(red stick) in the in silico phosphorylated structure (1FQW_PO3-_ (orange)) replicate those reported in the BeF_3_^−^ crystal structure (1FQW.pdb), The D57-PO_3_^−^, like BeF_3_^−^_,_ contributes to Mg^2+^ ion coordination, replacing a bound water molecule. The bond arrangement of the basal structure (2CHE (cyan)) is superimposed. B. Rotamer angle and bond distance distributions of activation indicators. Two sidechain ((i) Y106, (ii) T87) rotations and four bonds ((iii) K109-D57, (iv) N59-D57, (iv) T87-D57, (vi) Y106-E89)) distinguish the basal (cyan) and phosphorylated (orange) configurations. The mean values (± s.e) show the differences between the two configurations are greater than the spread of their individual distributions. The error bars (+/−) / bin show the variation between replicates. Residue label subscripts denote bonding atoms. C. Phosphorylation strengthens indicator couplings. The dynamic couplings between the six indicators are reported as a heatmap matrix of the mean nMI, corrected for the finite-size error. Values below and above the diagonal values are for 2CHE and 1FQW_PO3-,_ respectively_._ B and C are based on 3 × 4 ns replicates for each configuration. (For interpretation of the references to colour in this figure legend, the reader is referred to the web version of this article.)

MD simulations from 2CHE, and 1FQW_PO3-_ as starting structures recorded the variations (Fig. 1B) and the couplings between these parameters, reported by the normalized mutual information (nMI) (Fig. 1C). The differences between the basal and phosphorylated configurations were maintained, with an increase in mean coupling strength upon phosphorylation (2CHE nMI (0.07 ± 0.01); 1FQW_PO3-_ nMI (0.09 ± 0.01)). T87-D57 bond formation resulted in the most pronounced nMI increase in the dynamic couplings mediated by phosphorylation.

#### 2.1.2 Fold variation in the RR REC domain

We explored the relation of fold to function, by simulation as well as multi-step clustering of the available databases. We ran long (1 μs) simulations of the 2CHE and 1FQW_PO3-_ structures as well as the structure of the Mg^2+^-minus apo-CheY (3CHY) (Volz and Matsumura, 1991). The variations between replicates were assessed with PC1-PC2 plots (Supplementary Fig. S1). The relatively isotropic 3CHY PC1-PC2 plot became more anisotropic in 2CHE and 1FQW_PO3-_ as Mg^2+^ chelation allowed CheY to access more diverse conformational states. Next, we concatenated the 2CHE, 3CHY, and 1FQW_PO3-_ replicate trajectories. The PC1-PC2 plot of the concatenated 2CHE and 1FQW_PO3-_ trajectories revealed that overlap was restricted to a distinct C^α^ fold subspace (Fig. 2A). Cluster analysis of the concatenated trajectory identified 7 distinct clusters comprising 2CHE alone (*n* = 4), 1FQW_PO3-_ alone (*n* = 3), and mixed 2CHE-1FQW_PO3-_ (*n* = 1). Chelation affects the CheY fold since apo-CheY (3CHY) formed a distinct cluster (Fig. 2B). Thus, the basal and phosphorylated configurations have distinct C^α^ conformational landscapes, but also substantial overlap.

**Fig. 2:**
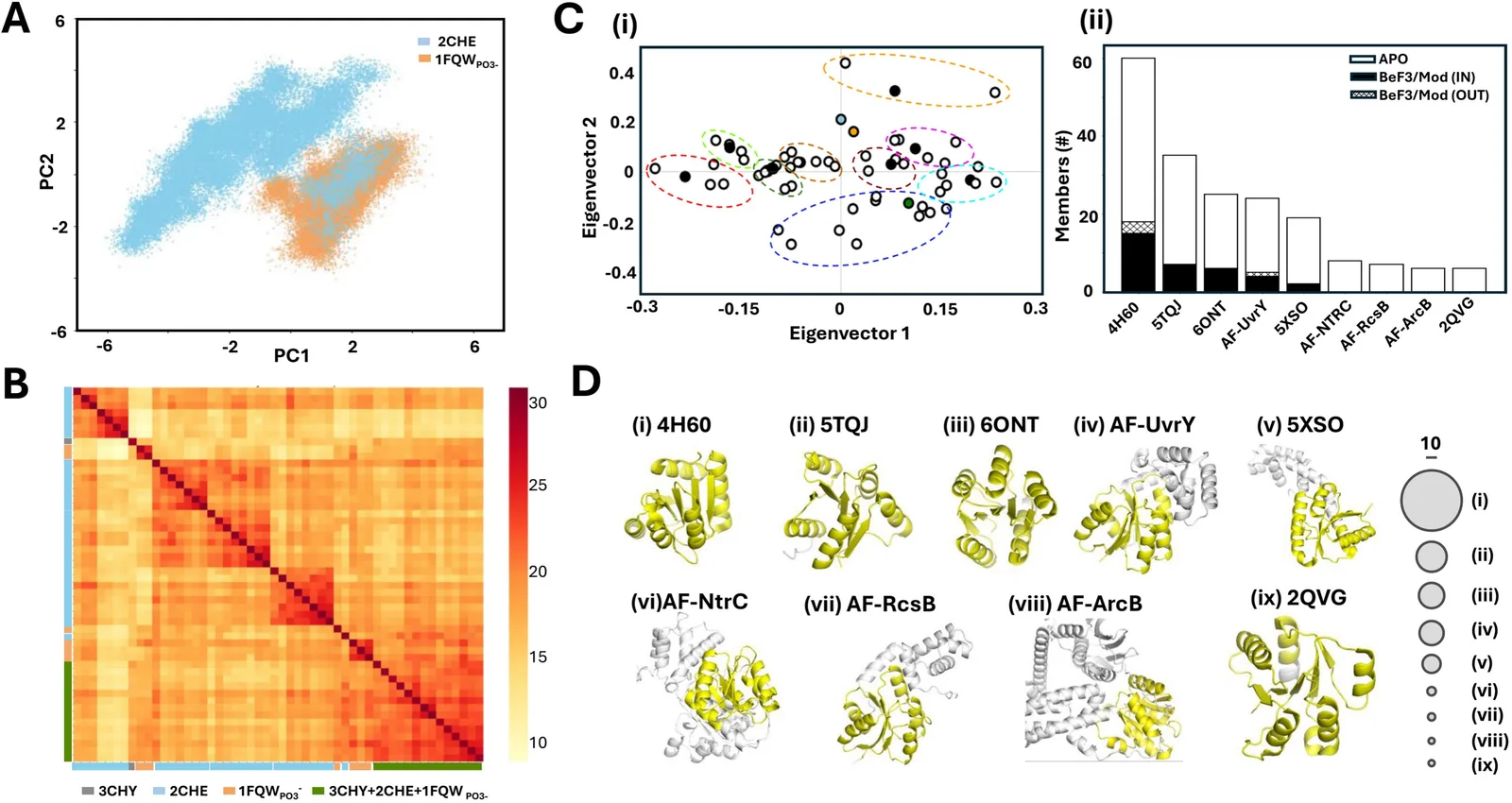
Conformational discrimination between basal and active configurations. A, B. MD simulations. Basal (2CHE (cyan)) and active (1FQW_PO3-_ (orange)) CheY_es_ configuration. 3 replicate 1 μs simulations per configuration. Intra-replicate variation is shown in Supplementary Fig. S1 for the apo (3CHY) as well as the native, basal configurations. A. PC1-PC2 plot of the principal backbone (C^a^) motions. B. The MD trajectories were concatenated and clustered (*n* = 64) in GROMACS. The DALI heatmap of the 64 cluster representatives shows distinct homogeneous 2CHE. 1FQW_PO3-_ and mixed (2CHE + 1FQW_PO3-_ (green)) groups. Apo (3CHY) configuration (gray). C, D. RR databases. C. (i) The DALI correspondence plot of the 59 representative REC domains (basal (open), active (black) after CD-Hit clustering of the 192-structure database. 2CHE (cyan), 1FQW_PO3-_ (orange). The 59 representatives were grouped with a K-means clustering algorithm (ellipses with dashed outlines) and screened for FunFam overlap. (ii). The distribution of all 192 REC domains among the final 9 clusters obtained after the FunFam screen. The fraction of structures modified by BeF_3_^−^ / other chemical modification or mutation are represented by the (black (Y106 IN)) and stippled (Y106 OUT)) bars. D. Left: The RR structures of the 9 group representatives (panels (i) – (ix)). The REC domains are colored yellow. Right: The gray circle diameters indicate group ((i) – (ix)) size. (For interpretation of the references to colour in this figure legend, the reader is referred to the web version of this article.)

Next, we examined REC domain conformational variation in the RR superfamily databases. AlphaFold (AF) models of entries in the seed PF00072 multiple sequence alignment (MSA), where available, were downloaded from InterPro or constructed de novo. The AF models were merged with the PDB crystal structures (< 2 Å resolution). The merger resulted in 192 non-redundant structures. Individual REC domains were extracted from this population. The 192 domains were filtered down to 9 structures in two steps (Fig. 2C). First, CD-Hit parsed the 1D sequence diversity, resulting in 59 clusters. Second, DALI-based correspondence analysis resolved the 59 cluster representatives into 11 groups as assessed by a K-means clustering algorithm. Two groups were merged based on a FunFam functional similarity screen of the CATH. database (Sillitoe et al., 2021) resulting in the assignment of the 192 domains into 9 groups (see Methods for details). The domains, modified with phosphoryl analogs or activating residue substitutions and checked for inward Y106 orientation as a diagnostic of activation, partitioned proportionally to cluster size, consistent with a random distribution insensitive to conformational variation.

The structural representatives of the 9 groups are shown in Fig. 2D. The representative of the dominant group is the structure of a *Vibrio cholerae* (*V. cholerae*) CheY4 protein, 4H6O (Biswas et al., 2013). There are 4 other crystal structures in the set, namely 2QVG, 5TQJ, 6ONT, and 5XSO, that are transcription factors. The remaining representatives are AF models of transcription factors. AF-UvrY, AF-RcsB, and AF-ArcB have the REC domain embedded within multi-domain proteins. AF-NtrC is a homodimer that oligomerizes to form higher-order assemblies. These representatives reflect the dominance of transcription factors as a functional group within the RR superfamily (Gao et al., 2019).

**Discussion:** Simulation duration as short as 4 ns are sufficient to reveal differences between the basal and phosphorylated configuration in salt-bridge formation and sidechain rotation in line with the Y-T coupling reported for BeF_3_^−^-modified CheY_es_ (Hastings et al., 2003; Lee et al., 2001a). The modest nMI couplings are consistent with the absence of a concerted switching mechanism suggested by published NMR relaxation dispersion experiments (McDonald et al., 2012) and MD simulations (Ma and Cui, 2007).

The survey of the InterPro and PDB databases reaffirmed that the static snapshots provided by crystal structures and AF models cannot separate the basal and activated conformations based on the variations in C^α^ backbone dynamics coupled to Y106 rotation states. The databases reflect the multiplicity of binding targets for diverse functions, consistent with previous surveys (Gao et al., 2007). Better discrimination was obtained between the unphosphorylated and phosphorylated forms of CheY_es_ by 1 μs MD simulations. The conformational landscape accessed by the apo-CheY form was more limited than for the Mg^2+−^bound forms consistent with previous evidence (McDonald et al., 2012; McDonald et al., 2013). Importantly, the simulations supported conformational selection as a dominant feature of CheY_es_ activation ((Wheatley et al., 2020) and references therein).

### 2.2 REC residue energy frustration and H-bond propensity

#### 2.2.1 Energy frustration in the REC domain

Energy frustration reports free-energy scores for the native contacts relative to the decoy distribution obtained by in silico substitution of all amino acids for each contact residue. Low residue scores indicate evolutionarily stabilized contacts, whereas high scores indicate the contacts are maintained far from the free energy minima. The frustration profile for the 59 post-CD-Hit structures were mapped onto the individual sequences in the 1D MSA and compared against the HMM logo for sequence conservation (Fig. 3). The superposition with the secondary structure profile revealed that (a) the minimally frustrated MSA positions map onto the central, predominantly hydrophobic β sheet. (b) The aspartate triad (D12, D13, D57) is maximally frustrated. D38, D64 and K109 have elevated maximal and neutral FrustIC scores. FrustrameterR (Parra et al., 2016) reports the D38, D64 and K109 residue positions form water-mediated bonds, based on separation and solvent-accessible surface area (SASA). R18, T87, Y106, K122, together with P82, G52, G65, G102, G104 have elevated neutral FrustICs. The bonding interactions of the D12, D13, D57 triad with metal ion, water, and other residues were explored to better understand the mechanistic basis for their energy frustration.

**Fig. 3:**
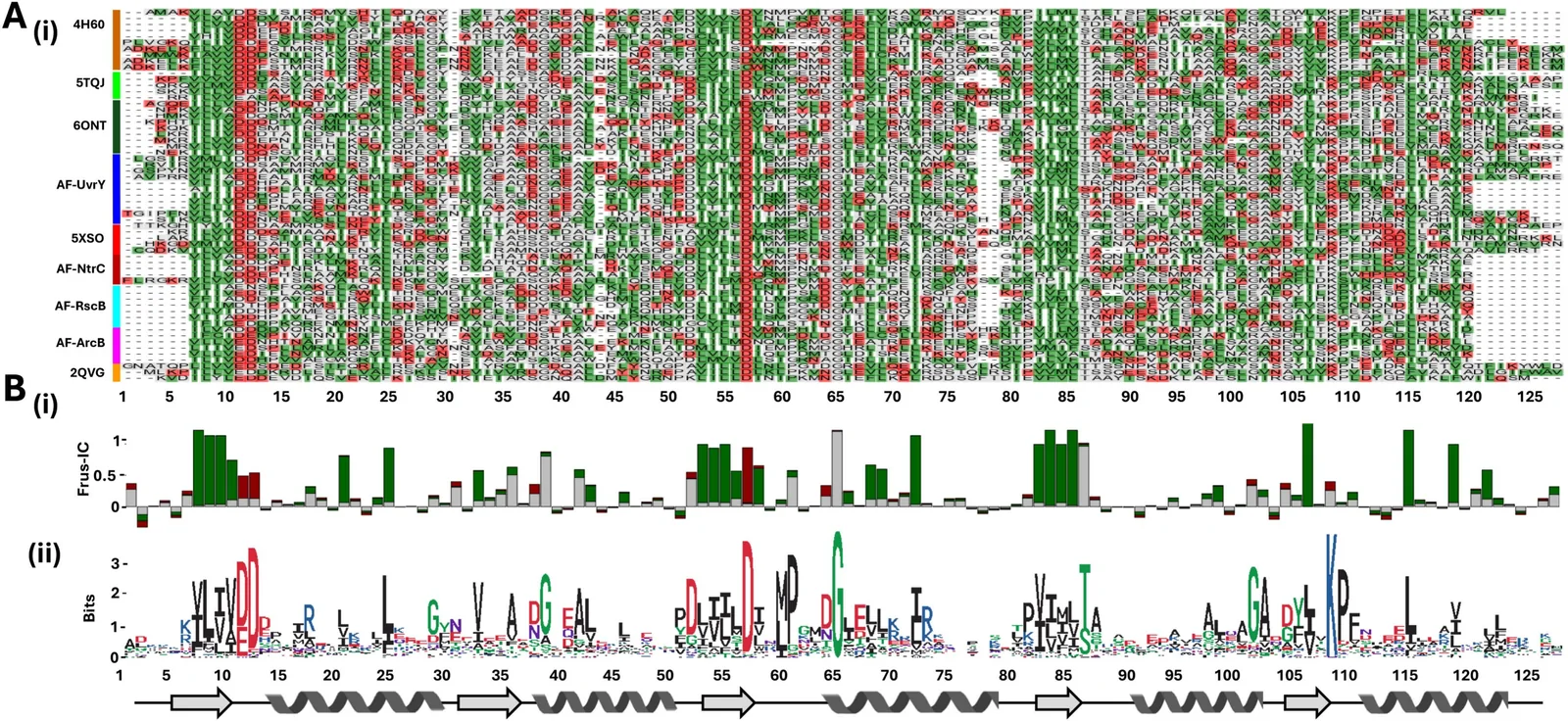
Frustraevo segregates the REC domain to a stable core and stressed phosphorylation site. A. Frustraevo REC domain MSA of the 59 CD-Hit cluster representatives, colored by FrustIC score (maximal (red), minimal (green), neutral (gray)). Bar (left) shows the assignment of the REC sequences to the final 9 groups (colour-coded as in Fig. 2 Ci). See Methods for FrustIC definition. B. FrustIC scores mapped on residue conservation and secondary structure. (i) The normalized FrustIC scores compared with (ii) the residue conservation HMM logo. Residue colour code (Parra et al., 2024). Secondary structure profile (a-helix (sinusoid), β-strand (arrow), loop (line)). Minimally frustrated residue positions map to the central β sheet. The aspartate triad in the phosphorylation site is maximally frustrated. (For interpretation of the references to colour in this figure legend, the reader is referred to the web version of this article.)

#### 2.2.2 Bound water at the CheY_es_ phosphorylation site

The (2CHE (1.8 Å), 1FQW (2.4 Å)) crystal structures have visualized water in metal cofactor coordination and H-bond rearrangements within the phosphorylation site. Nevertheless, the unknown functional roles of the majority of the localized waters add to concerns (Prisant et al., 2020) regarding water assignment in medium-resolution structures. Therefore, we ran MD simulations to compare the dynamics of localized waters to bulk solvent water based on their residence times and bond occupancy.

The Mg^2+^ coordination in the presence and absence of aspartyl phosphate for the energy-minimized 2CHE (basal) and 1FQW_PO3-_ (phosphorylated) structures is shown in Fig. 4Ai, ii, respectively. The 2CHE Mg^2+^ bonds with the D12, D13, D57 triad (Stock et al., 1993). The Mg^2+^ is coordinated with 2 water shells, as reported. The 1st water shell, closest (1.9 ± 0.0 Å) to the Mg^2+,^ was reduced to two in 1FQW_PO3-_, with one of the waters replaced by the phosphoryl group. The oxygen atoms of the 1st shell waters had the lowest B-factors in the published 2CHE structure. The more distant (29.1 ± 1.1 Å) three 2nd shell waters (*n* = 3) had higher B-factor values.

**Fig. 4:**
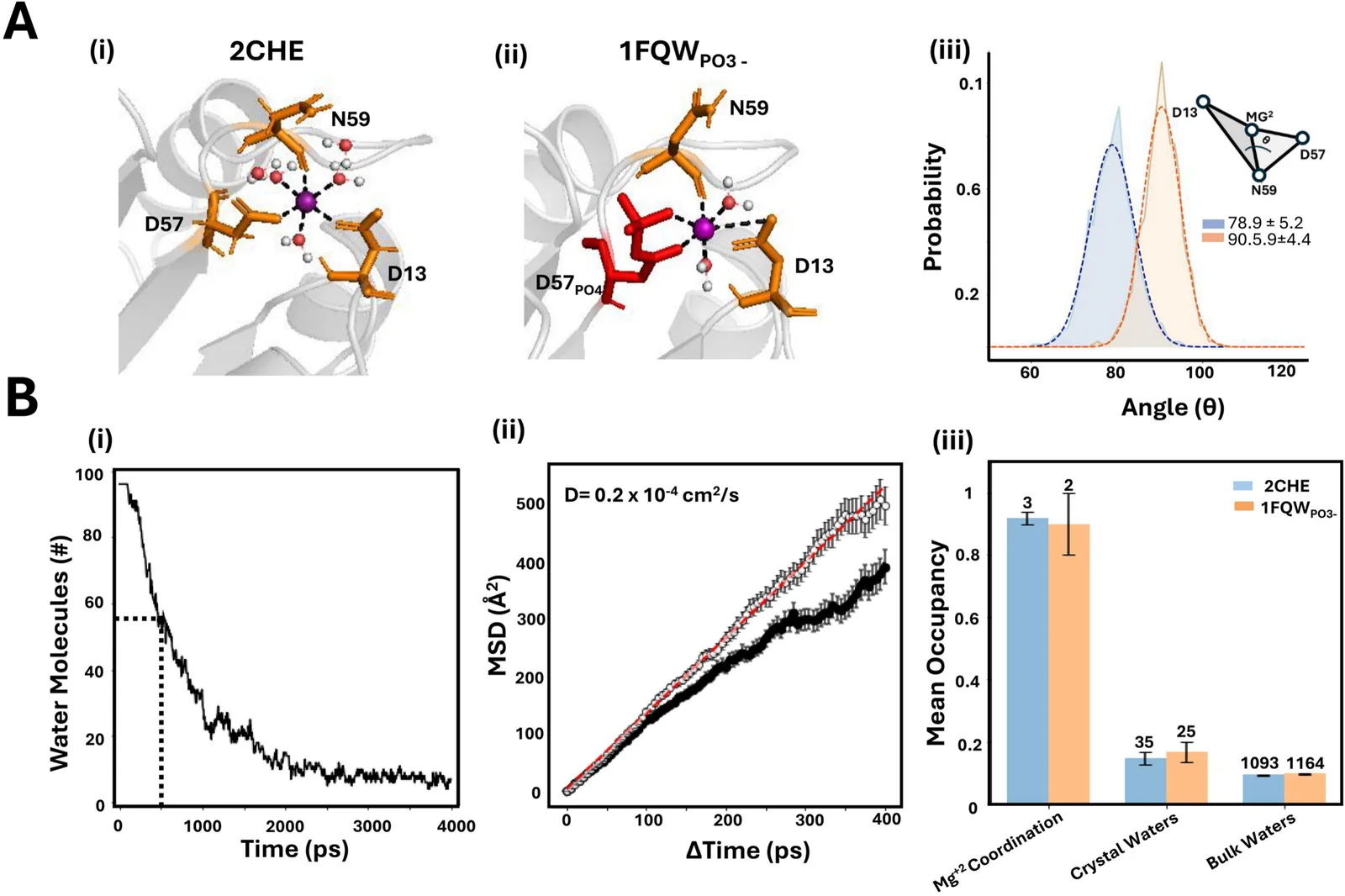
Bound water in the phosphorylation site and protein matrix. A. Mg^2+^ coordination. Snapshots of the initial (i) basal (2CHE) and (ii) phosphorylated (1FQW_PO3-_) configurations. Mg^2+^ (magenta). Water (oxygen (red), hydrogen (white)). (iii) The distributions and mean values (± s.e) of the divergence angle (θ) between the planes formed by the Mg^2^ with the C^α^ atoms of D13, N59, and D57, N59 for the 2CHE (cyan) and 1FQW_PO3-_ (orange)coordination topology are shown (3 4 ns replicates). B. Water – protein interactions. (i) The drift of the 2CHE crystal waters out of the water box minus CheY_es_. Half of the population exited in 500 ps (dotted lines). (ii) Mobility of crystal waters in the presence (hindered (black circles)) and absence (unhindered (white circles)) of CheY_es_. Linear best fit (MSD = 1.3 (Δ(Time)) (red line)) for the unhindered case (red line). D_unhindered_ = MSD/6(1.3(Δ(Time)) = 0.2 × 10^−4^ cm^2^/s. D_hindered_ = 0.15 × 10^−4^ cm^2^/s. (iii) H-bond occupancy (>0.05) of the Mg^2+^ 1st shell, crystal (minus Mg^2+^ 1st shell) and bulk waters. Numbers above bars give the population size. The 2CHE Mg^2+^ 2nd shell coordination water occupancy (0.01) was below the threshold (0.05). Bridge2 results from 4 ns trajectories (3 replicates). Supplementary Fig. S2 has additional details (see text). (For interpretation of the references to colour in this figure legend, the reader is referred to the web version of this article.)

MD simulations from these starting structures reported a slight, but persistent, difference between their Mg^2+^ coordination geometry (Fig. 4A.iii). In the absence of CheY protein, the crystal water population had a half-life of 500 ps within the water box. The mobility, computed from the mean squared deviation (MSD) versus Δ(time) plot, gave an apparent self-diffusion coefficient of 2 × 10^−4^ cm^2^/s. The value is in line with published values but affected by multiple parameters as discussed (Kadaoluwa Pathirannahalage et al., 2021). The diffusion coefficient was reduced in the presence of protein (Fig. 4Bi, ii). The displacement distributions of the 2nd shell waters showed a peak around 12 Å. We tracked the individual trajectories to understand this effect. We found that the residues that provide electrostatic solvation for the phosphorylation site form a local trap that slows the escape of the 2nd shell waters out of the pocket (Supplementary Fig. S2A, B).

Bridge2 reported the H-bond occupancy. The 1st shell waters in both 2CHE and 1FQW_PO3-_ were mostly fixed over the trajectory duration (4 ns). The 2CHE crystal water population occupancy was greater than that of non-crystal waters in the simulation box, even when the 1st shell waters were excluded (Fig. 4B). The residence time distributions of the protein-water (P—W) and protein-protein (P—P) H-bonds were comparable, but there was no correlation between the occupancy and the residence time for P—W H-bonds, in contrast to P—P H-bonds (Supplementary Fig. S2C, D). The published 2CHE residue B-factors were not correlated overall with the fractional occupancy computed from the MD simulations (Pearson correlation, *P*_cc_ = −0.21).

The residues that form P—W H-bonds are substantially fewer in number than the water molecules they interact with since the latter encompasses all waters in the simulation box. Transient H-bonds that resulted from high-frequency secondary structure fluctuations and interactions of surface residues with bulk water were removed with a persistence filter (> 15 ps). Charged and polar residues (*n* = 54) dominated this filtered population (*n* = 61), as shown in the 3D contact map (Supplementary Fig. S2E), with occupancies in line with those reported for other globular proteins (Qiao et al., 2019). The preference for residue type overrode the residue SASA.

#### 2.2.3 Residue H-bond propensity in representative REC domains

We could now study the role of the protein H-bond network in the activation of the REC domain having developed a protocol for identification of protein-water H-bonds. The betweenness centrality profiles of the CheY_es_ 2CHE and 1FQW_PO3-_ H-bond networks, were determined using the Bridge2 algorithm (Siemers and Bondar, 2021) following additional filtration by occupancy (>0.05%) to extract residues with high H-bond propensity.

We determined C^α^ centrality profiles to assess whether the H-bond centrality tracked backbone motions. The C^α^ network centrality profiles based on the conformational entropy of torsional angles (McClendon et al., 2012) were validated with custom scripts (Methods). The C^α^ centrality of the sequence segments around D57 (phosphorylation site) and Y106 (binding surface) increased in 1FQW_PO3-_ relative to 2CHE, consistent with the changes due to phosphorylation (Fig. 1) (Fig. 5A**)**. The MutInf couplings up-regulated and down-regulated by phosphorylation were documented for successive 3-residue fragments (Fig. S1B).

**Fig. 5:**
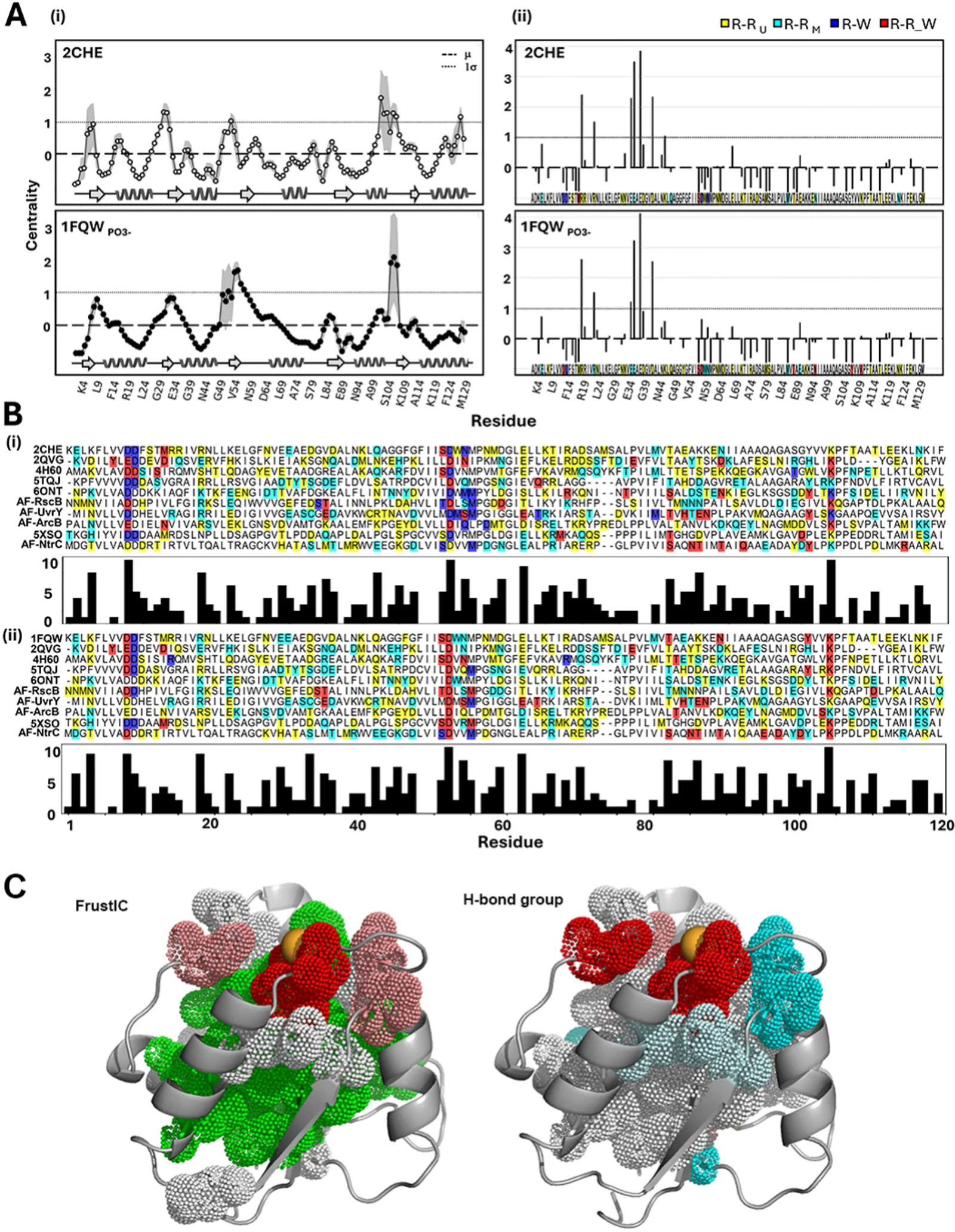
Comparative dynamics and residue H-bond states of the basal and active configurations. Extracted from 3 20 ns replicates. A. (i) The residue C^α^ backbone centrality (μ (open symbols) ± σ (gray error bars), Secondary structure profiles (α-helix (sinusoid), β-strand (arrow), loop (line)). (ii) The residue protein-water H-bond group (R-R_S_ (yellow), R-R_M_ (cyan), R-W (blue), R-R/W (red)) for the basal (2CHE) and phosphorylated (1FQW_PO3-_) CheYes states. B. H-bond group MSAs of RR superfamily representatives. (i) Basal and (ii) phosphorylated configurations. Central residue positions are coded by group as in A(ii) (top) and conservation (bottom). C. H-bond propensity compared with local energy frustration. (i) The minimally frustrated (green). maximally frustrated (red) and moderately frustrated (salmon (higher IC_MAX_) / white (higher IC_NEU_)) residue positions (dot representation). (ii) Corresponding H-bond group map for the same residue positions. H-bond group ((R-W + R-R/W) red), R-R_M_ higher (cyan), lower (light cyan fraction), None (white). Mg^2+^ (orange sphere). Statistics (Table 1), PSE structure files (Supplementary Material).. (For interpretation of the references to colour in this figure legend, the reader is referred to the web version of this article.)

The Bridge2 H-bond centrality localized to discrete residue positions that formed H-bonds and required a more nuanced analysis. The arginine-rich (R18-R22) and the acid-rich (N31-D41) segments in both basal and phosphorylated configurations. We partitioned the central residues into four groups: (i) residue-residue H-bonds (R-R_S_), (ii) residue-water H-bonds (R-W), (iii) switchable H-bonds that bonded alternately to multiple residues (R-R_M_) or alternately (iv) with water or residue (R-R/W). The partitions allowed the malleable R-W, R-R/W, R-R_M_ residue positions to be tracked individually or collectively as appropriate.

Bridge2 was subsequently applied to equivalent MD runs (20 ns, 3 replicates) to the other nine representatives of the RR superfamily and the H-bond group assignments (*n* = 61) presented as an MSA with columns identified by the CheY_es_ residue (Fig. 5B). We computed the background group probabilities to evaluate the significance of the observed probabilities at residue positions of interest and examined the correlation between the Frustraevo and H-bond group MSAs in the two sets of interest identified earlier - the maximally frustrated phosphorylation site (D12, D13, D57, K109 – Set-1) and the moderately elevated ${\mathrm{FrustIC}}_{\mathrm{Comb}}$ (R18, D38, D64, P82, T87, Y106, K122 - Set-2) minus glycine residue positions (Table 1).

**Table 1:** *t*-test and Pearson correlation coefficients. The *t*-test probabilities (P_T.test_) report the likelihood that the difference between the observed (F_obs_) and background (F_bkd_) H-bond group frequencies is significant. The water groups were combined (R-W, R-R/W) to better assess the water contribution. F_bkd_^(R-W, R-R/W)^ = 0.064, F_bkd_^(R-Rm)^ = 0.094. F_bkd_^(R-R)^ = 0.15. The Pearson coefficients (Pcc) compare the H-bond group F_obs_ frequencies with the combined FrustIC (FrustIC_comb_) scores. Significant correlations are highlighted by H-bond group colour. See text for the maximal (Set-1) and intermediate (Set-2) frustration sets of residue positions. The R-W, R-R/W groups had higher normalized SASA (0.37) than R-R_S_, R-R_M_ (0.25). The difference was significant (*p* = 0.0003 (two-tailed *t*-test)). The F_obs_ (0.8 ± 0.06) for no H-bonds was above background (F_bkd_ = 0.69) for the minimally frustrated positions. The combined ${\text{FrustIC}}_{\text{Comb}}$ score was (−0.24 ± 0.04) for all versus (−0.95 ± 0.05) for the minimally frustrated residue positions.

SET	TEST	R-W + R-R/W	R-RM_)_	R-R
**1**	**P**_**T.test**_**(F**_**obs**_**- F**_**bkd**_**)**	**0.0004**	0.27	0.011
	**P**_**cc**_**(FrustIC**_**comb**_**vs F**_**obs**_**)**	**0.88**	−0.72	−0.59
**2**	**P**_**T.test**_**(F**_**obs**_**- F**_**bkd**_**)**	0.089	**0.011**	0.113
	**P**_**cc**_**(FrustIC**_**comb**_**vs F**_**obs**_**)**	−0.2	**0.24**	0.28

These correlations between the Frustraevo and H-bond group MSAs were illustrated by their superimposition on the 2CHE map (Fig. 5C). The minimally frustrated residue columns (*n* = 22 (7 V, 7 L, 3I, 2F, 1 W, 1S, 1 M in 2CHE) comprised mostly hydrophobic sidechains mapped to the protein core. H-bond formation in this group was rare and insufficient for statistical analysis.

**Discussion.** The algorithms for energy frustration have studied water-mediated bonds from their earliest inception to establish a correspondence between minimal energy frustration and the hydrophobic core (Ferreiro et al., 2007). We now extend the correspondence between minimal frustration and the hydrophobic core to the RR family. The aspartate triad and K109 are maximally frustrated. The observed frustration is consistent with recent work based on the AWSEM force field that has shown patches of maximally frustrated residues localize at catalytic (Freiberger et al., 2019) and metal coordination (Chen et al., 2020) sites.

The measured bulk water densities and diffusion coefficients, in line with published estimates, validated the simulations of water dynamics. The water molecules for Mg^2+^ coordination had extended lifetimes (> 1 ns) relative to the protein-water H-bonds lifetimes (10 - > 1000 ps). The occupancy difference between the H-bonds formed with the protein by localized crystal waters versus waters added to the simulation box was slight, with H-bond propensity determined by the protein residues. There was a strong correlation between the frustration (${\mathrm{FrustIC}}_{\mathrm{Comb}}$) and the water H-bond propensity of residue set-1 (D12, D13, D57, K109), but the propensity cannot be the sole determinant of maximal frustration. The S56 residue position has water H-bond propensity comparable to D13, but minimal frustration. Notably, it does not participate in either Mg^2+^ coordination or phosphoryl transfer.

The results for Set-2 (R18, D38, D64, P82, T87, Y106, K122) are more nuanced. R18 and D38 are important for the solvation of the phosphorylation site (Hamid et al., 2022), T87 and Y106 switch orientation upon phosphorylation (Fig. 1), while the D64 column H-bond group distribution in the representative RR MSA (Fig. 5b) is biased towards the switchable R-R_M_ H-bond group. The P82, as well as the excluded glycine, positions localize to flexible loop elements. These residue positions are unified by their relevance for conformational plasticity, but the arguments to relate plasticity to frustration (Parra and Ferreiro, 2025) remain speculative.

### 2.3 Network construction and analysis

#### 2.3.1 The physical CheY_es_ H-bond network.

We constructed the H-bond networks for the two representatives of the two major RR sub-families (Fig. 2Cii), namely 4H6O and 5TQJ, in addition to CheY_es_. The node connectivity was represented by the residue centrality. The edges represented the temporal coupling between nodes, reported by the H-bond occupancy. The bonds were physically constrained by distance and angle. The networks (Fig. 6) provided a more sensitive discrimination than the centrality profiles of the basal and phosphorylated configurations (Fig. 5a.ii). The 4H6O residue positions are renumbered to match the CheY_es_ positions in the MSA (Fig. 5B) for easier comparison between these chemotaxis RRs (Fig.S3A).

**Fig. 6:**
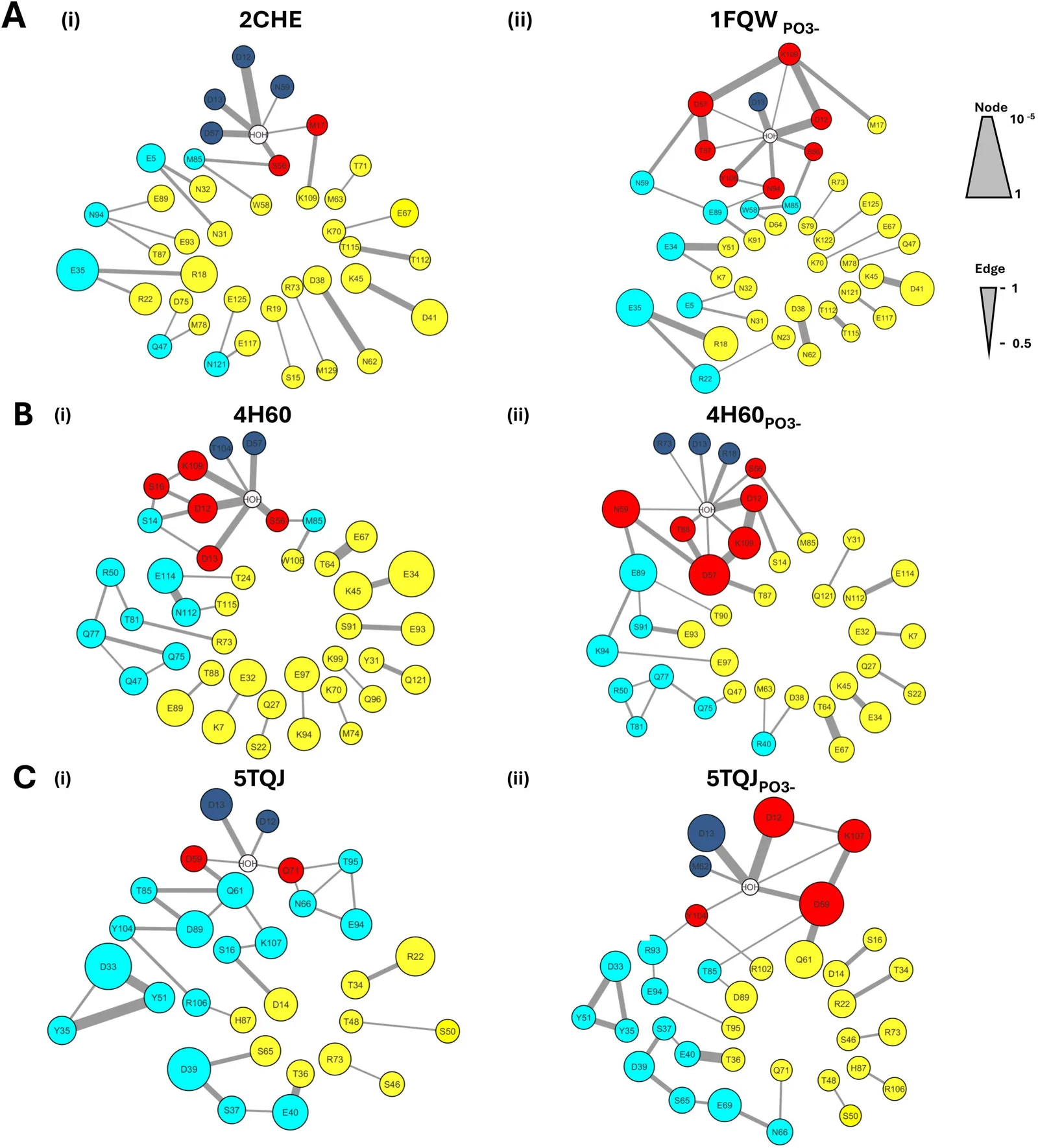
H-bond networks of representative RR REC domains. Residue nodes are colored by group, as in Fig. 5Aii. Node diameter indicates centrality; edge thickness between nodes the bond occupancy. Pyramidal keys give values. Node size 1 = centrality 10^-3. Cumulative 3x20ns replicates per configuration. (A) CheY_es_ (i) basal (2CHE), The water-mediated (R-W and R-R/W) residue nodes in 2CHE are (D12, D13, M17, S56, D57), with K109 and M85, W58 bonded to M17 and S56 respectively. High occupancy, non-water R-R_S_ and R-R_M_ H-bonds connect N31, N32 with E5, R18, and R22 with E35, D38-N62, D41-K45. (ii) In phosphorylated 1FQW_PO3-_ T87, N94, Y106, K109 are added to (D12, D13, S56, D57) with M17 bonded to K109. High occupancy H-bonds are formed between D57-T87, D57-K109, and K109-D12. D57 is chained to Y106 and K91 via N59, E89 and N94. The E35-R18, E35-R22, D38-N62, and D41-K45 high occupancy H-bonds are retained, with the addition of E35-Y51. (B) 4H60 (i) basal. (ii) phosphorylated. (C) 5TQJ (i) basal (ii) phosphorylated. The phosphorylation-triggered changes in network architecture follow a similar trend to CheY_es_. (B) Phosphorylated, but not basal, CheY4_vc_ D57 forms high occupancy H-bonds with T87, K109, and N59. It connects, via N59, E89 to S91, K94, and E97 in helix α4. W106 (CheY_es_ Y106) is not part of either the basal or phosphorylated H-bond network. The R-Rs T E34-K45, T64-E67 H-bonds stabilize the CheY4_vc_ network. S91–93, N112-E114 switch between the R-R_S_ and R-R_M_ groups upon phosphorylation. Renumbered residue positions (see text). (C) Phosphorylated, but not basal, 5TQJ D57 H-bonds with T87, K109, and Q59 (CheY_es_ N59). Basal 5TQJ D57 is not isolated, but connects via Q59 with T87, Y106, and D91. 5TQJ D57-Y106 connectivity is lost upon phosphorylation, The high-occupancy 5TQJ R-R_M_ H-bond networks (D34-Y36-Y51, T36-R22, T37-E41-S38-D40) are not responsive to phosphorylation. High occupancy D12-K109 H-bonds are formed in both phosphorylated CheY4_vc_ and 5TQJ.

The CheY_es_ D57 network centrality and connectivity are increased upon phosphorylation. The D57-PO_3_^−^ connects with the interfacial Y106, K91 via multiple pathways absent in the basal configuration. The conduits are mediated by N59 and E89, with the connection to Y106 also involving D94. Strong H-bonds of D57-PO_3_^−^ with K109, T87; and K109 with D12 are also formed. H-bonds formed between R-R_S_ residues were dedicated to fold architecture. They did not contribute to chained relays or alternating partner transitions important for network dynamics (Fig. 6A).

The multiple H-bonds formed by D57-PO_3_^−^ and K109 are an important feature of the increased connectivity. We reasoned that the multiple bonds could reinforce their collective H-bond occupancy. We evaluated this idea by studying the H-bonds formed by the D57-PO_3_^−^, W58, T87, Y106 and K109 as these are prominent determinants of the phosphorylated configuration.

We will use “*H-bond*_*MAX*_” for the maximum number of H-bonds formed by a residue. The number of bonding states per residue will increase combinatorially as *H-bond*_*MAX*_ increases, given that residues can simultaneously form H-bonds with multiple partners. Snapshots of the *H-bond*_*MAX*_ states for these residues are shown in Fig. 7. K109 H-bonds with M17 in the basal configuration, but binds D12 and D57 upon phosphorylation to expand the phosphorylation site together with T87. The T87 and Y106 sidechains reorient into the hydrophobic core upon phosphorylation to H-bond with internal water distinct from the bulk solvent. The W58 indole group can bridge between β4 (D64) and β5 (M38) when reoriented by phosphorylation. It provides a mechanistic explanation for the role of W58 in the allosteric network reported in the literature (Introduction).

**Fig. 7:**
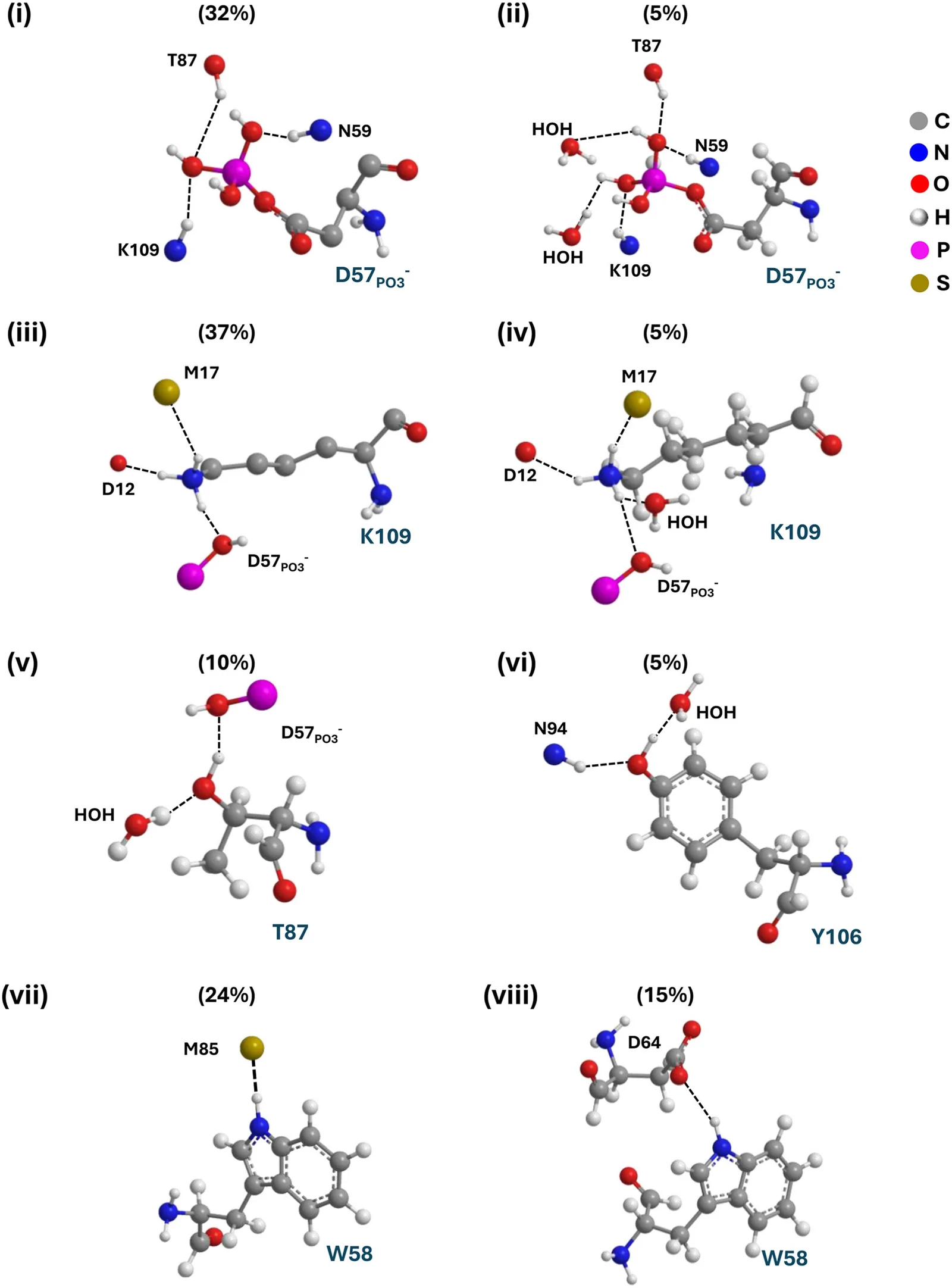
1FQW_PO3-_ phosphorylation increases the H-bond_MAX_ of key residue H-bonds. D57_PO3-_ (i (32%), ii (5%)). Water H-bonds, albeit rare, can increase H-bond_MAX_. K109 (iii (37%), iv (5%)). The K109 ε-amino nitrogen has high H-bond propensity. It can H-bond simultaneously to D13 and M17, as well as the D57_PO3-_ phosphoryl. In addition, it can add an H-bond with water. The mean K109-M17 persistence time in the panel (iv) state is short (< 15 ps). T87 (v (10%)). T87 H-bonds with water simultaneously with D57_PO3-_ in the high dielectric environment of the phosphorylation site. Y106 (vi (5%)). Y106 rotates inwards upon phosphorylation to bond to N94. Its sidechain phenol bonds to water. W58 panels show weak H-bonds formed alternatively by its indole ring with the M85 sulfur (vii (24%)) or the D64 carboxyl oxygen (viii (15%)). These 1FQW_PO3-_ states, except the W58-M85 H-bond, were not observed in 2CHE simulation runs. Instead, 2CHE D57 bonds with water (61%), K109 with M17 (32%), and T87 with N94 (12%). Brackets show percentile occupancies. Panels (ii, iv, v, vi) show bonded waters. ChemDraw illustrations.

#### 2.3.2 Comparisons between CheY_es_ and other REC H-bond networks

4H60 is the crystal structure of *V. cholerae* CheY4 (CheY4_vc_)). It represents the largest group that includes structures of chemically modified phosphoryl mimics as well as intraspecies paralogs. Its crystal structure shows an altered target binding surface with W101, equivalent to CheY_es_ Y106, embedded in the hydrophobic core in both the basal and BeF_3_^−^-modified states. CheY4_vc_ does not bind FliM (Biswas et al., 2013). Therefore, the comparison between CheY_es_ and CheY4_vc_ was of particular interest, given the experimental evidence for the differences in Y106 sidechain orientations and binding to FliM.

5TQJ is the crystal structure of a *Burkholderia phymatum* transcriptional regulator that belongs to the LuxR RR sub-family (Tsai and Winans, 2010). It may dimerize via the C-terminal α4-β5-α5 face, as in the *E. coli* OmpR/PhoB ArcA (Toro-Roman et al., 2005) or helix α6 as in the *Pseudomonas aeruginosa* LasR (Bottomley et al., 2007) transcriptional regulators. 3D-superimposition has shown these dimerization surfaces overlap or are proximal to the CheY_es_ binding interface. We documented the 5TQJ H-bond network to provide a window on allosteric regulation in the dominant family of RR transcriptional regulators, even though its biochemistry is uncharacterized.

The increased connectivity of the phosphorylation site is the central feature of the H-bonded networks for all three RRs (Fig. 6). CheY4_vc_ D57, D12, D13 and N59 H-bond with water in the basal configuration, D57 becomes the prominent node, strongly connected to T87, T88 and K109 upon phosphorylation, The CheY4_vc_ D57_PO3_^−^ also connects to the interfacial T90, S91 and K94 via N59 and E89, as in CheY_es_, but not to W106. The phosphorylation-dependent changes in network node centrality (CheY_es_ D12, D57, N59, N94, K109; and equivalent 4H60 nodes) are significant when compared to the variation across replicates (Supplementary Fig. S3B).

D57 is again strongly coupled in 5TQJ_PO3-_ relative to 5TQJ. Basal D57 is connected to Y106 via Q59, T87 and D91, but while the Q59 and T87 H-bonds remain, the Y106 connection is lost in the phosphorylated configuration. As in CheY_es_, D57_PO3_^−^ is the prominent node, with a strong bond between it and K109.

In all cases, the β2 strand forms H-bonds (CheY_es_ (E35 - > R18, R22, D38 - > N62, D41 - > K45), CheY4_vc_ (E34 - > K45), 5TQJ (D33 - > Y35, Y51, S37- > E40- > T36)) that are insensitive to phosphorylation. These bonds would stabilize the H-bond network. The predicted 5TQJ network model was not studied further in the absence of functional data or comparable crystal structures.

The physical H-bond network will have associated with it a dynamically coupled network that reflects the C^α^ backbone and sidechain dynamics tied to H-bond fluctuations. We compared the associated dynamic couplings in CheY_es_ and CheY4_vc_ to elucidate the differences between these single Che domains. We constructed the nMI matrices for these dynamic couplings between H-bond states for the residues reported by the Bridge2 centrality. The nMI value for each residue pair (R_x_, R_y_) was computed after removal of the H-bond contacts, if any, between them. The matrices were first filtered with a decoy distribution. Significant couplings were extracted from the residual distributions (Methods) and compared for the variation between replicates (Supplementary Fig. S3C).

The backbone and sidechain motions are illustrated in videos that compared the basal and phosphorylated CheY_es_ H-bond networks (Supplementary Fig. S4A). Each video is composed of snapshots that record the transitions observed for the “switchable” H-bonds formed between residues in groups ((iii) R-R_M_), ((iv) R-R/W) that form multiple H-bonds and switch binding partners (either residue or water) to increase the number of states. The videos provide the spatiotemporal detail to build on the representations of the 2D H-bond networks and *H-bond*_*MAX*_ states.

The matrices with the residual couplings are shown for both basal and phosphorylated CheY_es_ and CheY4_vc_ (Supplementary Fig. S4B). The D57_PO3_- *H-bond*_*MAX*_ was greater than D57, resulting in increased state multiplicity upon phosphorylation, with many significant couplings (Supplementary Fig. S4C). These figures, taken together, show that there is a general increase in the number of couplings, the coupling strength, and state multiplicity upon phosphorylation.

The dynamic couplings were mapped onto the 3D structures of the basal and phosphorylated forms of CheY_es_ and CheY4_vc_ (Fig. 8). The networks, unconstrained by distance, include significant long-range couplings with a mean distance that is typically thrice the H-bond distance (3.5 Å). Many couplings span the phosphorylation site to connect residues in the surrounding loops and α-helical segments. Inspection of the couplings reveals three categories. (a) H-bonded residue pairs with multiple H-bonds that remain coupled after removal of the bond between them (e.g., 1FQW_PO3-_ D57-N59, CheY4_PO3-_ D57-K109). (b) Distant residue pairs connected by a chain of H-bonds (e.g., 1FQW_PO3-_ D57-Y106, CheY4_PO3-_ D57-K94). (c) Unconnected distant residue pairs (e.g., 1FQW_PO3-_ D57-D38, CheY4_PO3-_ D57-R18). The H-bond-associated dynamic networks provide a complementary description of the change in the key couplings triggered by phosphorylation.

**Fig. 8:**
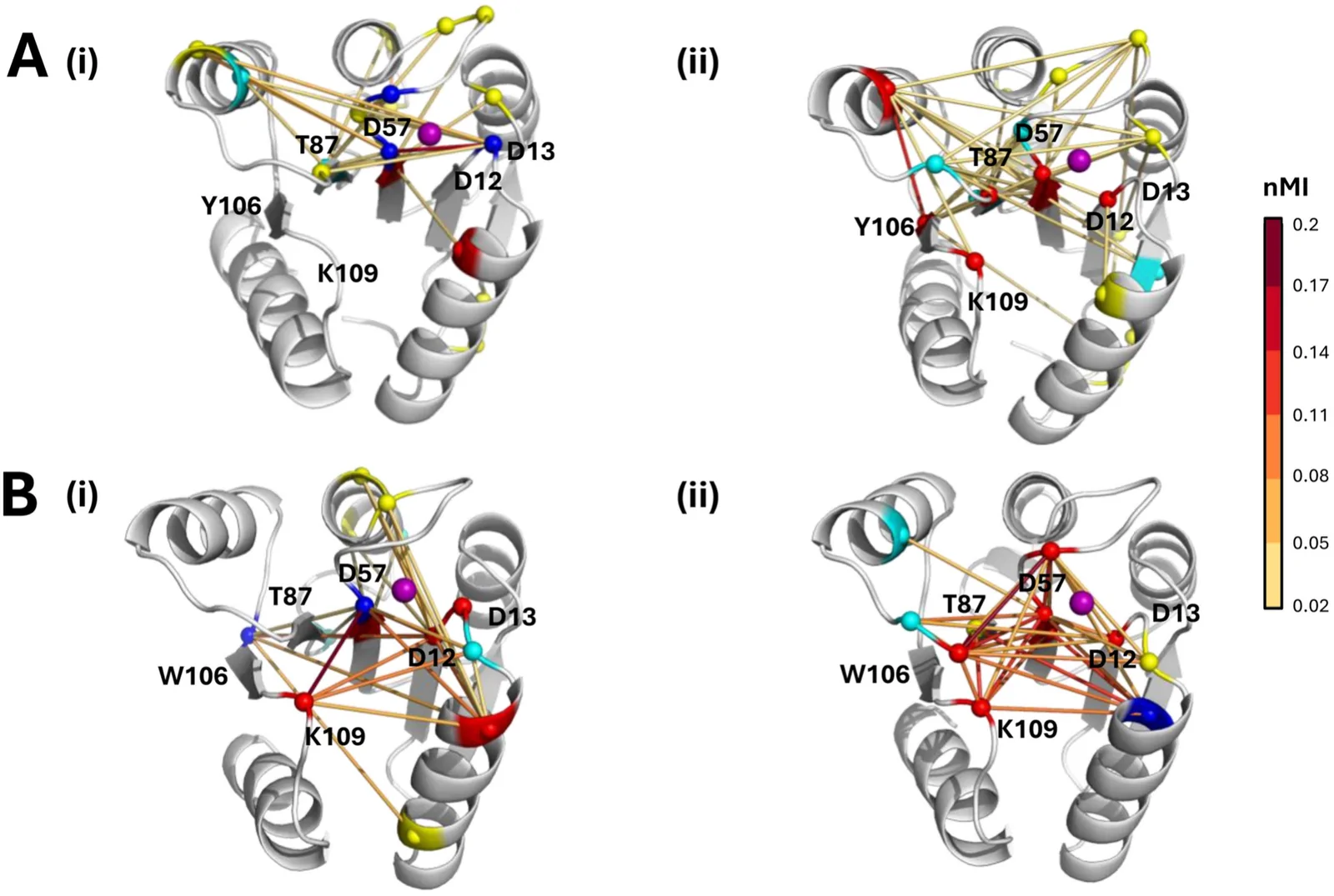
The associated long-range dynamic network. The nMI couplings mapped onto the 3D structures. A. CheY_es_ (i) basal (10.4 ± 1.1 Å), (ii) phosphorylated (13.9 ± 1.2 Å). B. CheY4_vc_ (4H6O) (i) basal (14.2 ± 1 Å), (ii) phosphorylated (9.5 ± 0.6 Å). The brackets show the mean (+ s. e) C^α^-C^α^ distances of the couplings. Line colour scales with nMI value. Residues are colored by H-bond group as in Fig. 5Aii.

A network of long-range couplings links CheY_es_ Y106 with D57-PO_3_^−^, with the most dominant relay mediated by N94. These nodes, together with T87, D38, and E89 dominate the long-range network. The T87 – E89 sequence segment transitions to a major dynamically coupled nexus upon phosphorylation. The CheY_es_ T87 is dynamically coupled to Y106 and residues localized around the phosphorylation site (R73, S79, N94). Thus, Y-T coupling while present, is not the sole determinant of the linkage between Y106 with D57-PO_3._ There are fewer couplings in the basal CheY_es_ network, and the Y106 and T87 nodes do not contribute (Figs. S3C, S4B).

The comparison of the CheY_es_ and CheY4_vc_ long range networks reveals the major difference is the absence of CheY4_vc_ W107 (CheY_es_ Y106), as in the direct H-bond network. This result implies that W107 motions do not affect the phosphorylation-dependent alterations of the free CheY4_vc_ target binding surface, in contrast to Y106 in free CheY_es_. There is a marked phosphorylation-dependent increase in the CheY4_vc_ K110 (CheY_es_ K109), T88 (CheY_es_ T87) couplings with the equivalent aspartyl phosphate and each other. The dynamic CheY4_vc_ D57-PO_3_^−^ linkage with T87 is mediated via N60, S57 (CheYes N59, S57) instead of N94 as in CheY_es_. It is remarkable that a similar set of a few residue positions is used to remodel both the CheY_es_ and CheY4_vc_ REC domains given the number of possible couplings between the residue population competent to form H-bonds.

**Discussion.** Aspartyl phosphate lifetimes depend on water, as well as other environmental factors (Cavanagh et al., 1994). Thus, a quantitative analysis of water H-bond dynamics to show the prominence of the D57-PO_3_^−^ node is of particular importance. The couplings reported by the CheY_es_ networks are supported by experimental evidence. N59 is one of the residues implicated in the steric block of D57 from solvent attack (Pazy et al., 2009) and has high solvent accessibility. (Hamid et al., 2022). The K91-E89 bond would immobilize the β4-α4 loop consistent with the reduced loop flexibility and solvent accessibility in the 13DK106YW phosphomimic (Wheatley et al., 2020).

The MD revealed rare multiple bond states not observed in the static snapshots provided by the crystal structures. These states have been reported in other proteins. There are examples where K109 can achieve an H-bond_MAX_ of 5H-bonds (Rogacheva et al., 2017). Hydrogen bonding of the threonine in the glycophorin A dimerization motif drives transmembrane helix association (Smith et al., 2002). The T87-D57_PO3-_ H-bond could play a similar role to associate the β-β strands in the CheY_es_ core. Tryptophan indole H-bonds are weak in a high dielectric, but the internalized W58 may communicate via the hydrophobic core to the interfacial surface, as documented for membrane proteins (Khemaissa et al., 2022). The Y106 hydroxyl H-bond with water could reduce the cost for insertion of the hydroxyl in the core (see (Pace et al., 2001)). The internal water is distinct from the bulk solvent, but its presence as well as the multiple bonding of the bulky tryptophan suggests that the core breathes.

The network architectures corroborate and add to the crystallographic evidence that CheY_es_ Y106 reorients upon phosphorylation, whereas CheY4_vc_ W107 does not. The direct couplings between D57-PO_3_^−^, K109 and T87 are supplemented by the long-range couplings mediated by intermediate nodes in the two REC domains. Mutations in the intermediate residues (S56, N59, N94) result in chemotactic lesions. The modification of the basal structures by PO_3_- affects a common set of core couplings across REC domains, and the differences are supported by experiment. They are unlikely to occur by chance.

CheY4_vc_ has been proposed to be a phosphate sink that competes with its motor binding homolog (CheY3_vc_) for the CheA kinase to tune the *V. cholerae* chemotactic response in line with other bacterial species (Amin et al., 2014). The idea is consistent with biochemical evidence that CheY4_vc_ does not bind FliM. It would be important to show that CheY4_vc_ binds the CheA kinase to support this proposition. In *E. coli*, CheY helix α4 is part of the CheA binding surface. The phosphorylated CheY4_vc_ H-bond network links helix α4 residues S92, E94, K95 (= CheY_es_ K91, E93, N94) with D57, whereas the unphosphorylated network does not. Crystal structures of the CheY4_vc_ complex would be decisive given the heterogeneity in binding modes between organisms (Park et al., 2004).

## 3 Conclusions

### 3.1 Highlights of the study

We used MD simulations to study the temporal H-bond dynamics that characterize the conformational landscapes of the basal and phosphorylated configurations of the REC domain in CheY_es_ and selected representatives of the RR superfamily. We elaborate the bullet-point highlights of our study below:

(1) We filtered by sequence, fold, and functional similarity for the most comprehensive selection of RR superfamily representatives. The representative clusters did not distinguish between basal or activated states, or between functional RR subfamilies. MD simulations of the basal and phosphorylated configurations of the representatives (Fig. 5), followed by the construction of network models (Fig. 5, Fig. 8), consolidated the knowledge of CheY_es_ allostery and extended it to two functionally diverse RR representatives of the two major clusters, as schematized in Fig. 9.

**Fig. 9:**
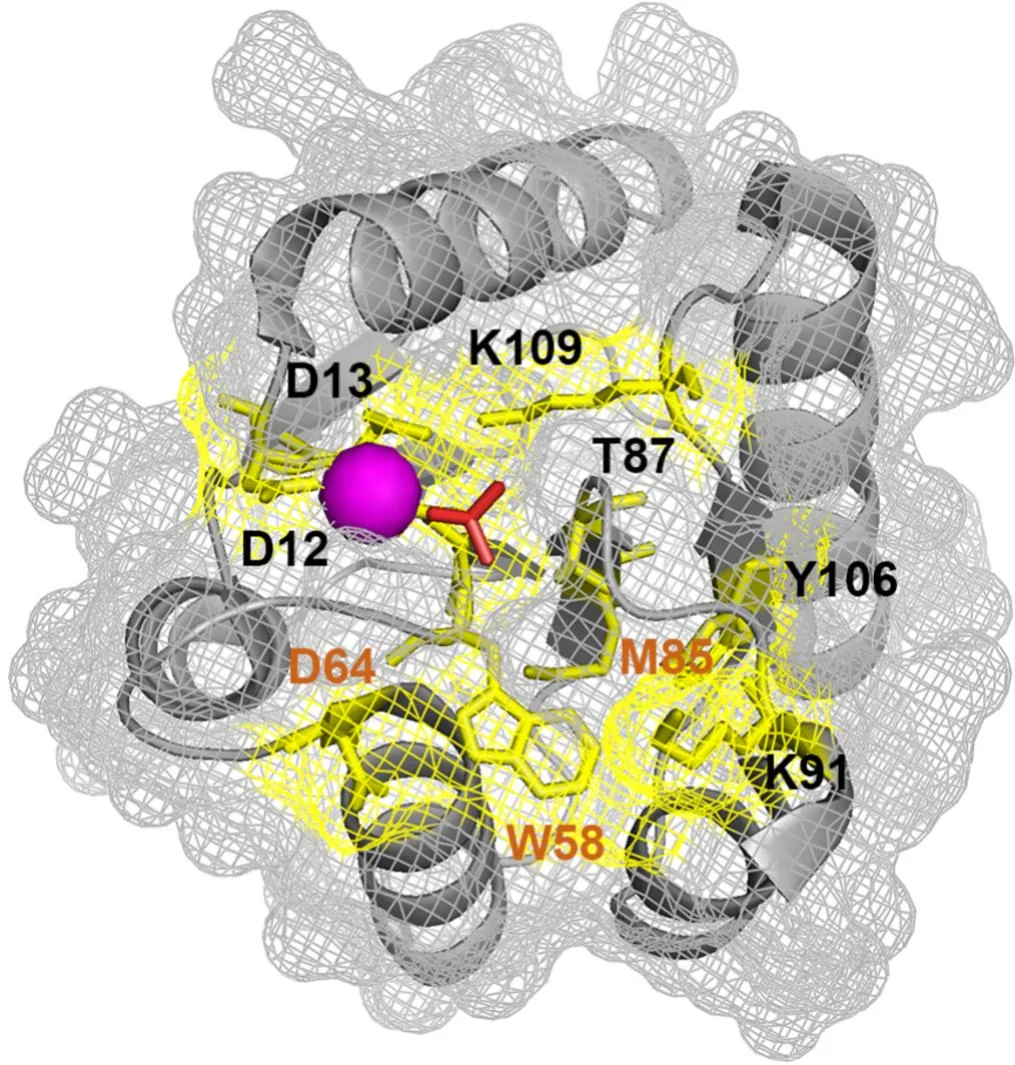
The CheY_es_ H-bond network. The role of H-bonds, including P—W bonds, revealed by this study is mapped (yellow mesh) onto the 1FQW crystal structure. The temporal dynamics of the Mg^2+^ (magenta sphere) coordination 1st and 2nd shells were determined. The water-rich aspartate triad (D12, D13, D57) and its H-bonds formed upon phosphorylation with the switch residues T87 and K109 are a common feature of the 3 RRs examined, but the associated long-range dynamic couplings, shown in Fig. 8 are variable. The chained H-bond connection between D57_PO3-_ and K91 (or equivalent residues) is replicated, but the CheY_es_, D57-Y106 chain is not in the other two RRs. The W58 bridge may also be a specific CheY_es_ characteristic. Label colour distinguishes residues that form water H-bonds (black) from ones that do not (orange). (For interpretation of the references to colour in this figure legend, the reader is referred to the web version of this article.)
(2) We characterized the role of H-bonded waters with Bridge2, a high-throughput H-bond network graph algorithm (Siemers and Bondar, 2021), and the Frustraevo package (Parra et al., 2024) that gives a residue-level readout of protein fold stability. Bridge2 was developed for the analysis of H-bonds in membrane proteins (Siemers et al., 2019), but it has been used to study the role of interfacial waters in protein assemblies (Karathanou et al., 2020). Its application to small, soluble proteins, such as the RR superfamily, is relatively novel. We extended previous insights on the minimal energy frustration for the hydrophobic core (Ferreiro et al., 2007) and maximal frustration for metal coordination in the RR superfamily (Chen et al., 2020). The correlation between frustration and the water H-bond groups can be explained by the fact that water at the phosphorylation site is required for PO_3_^−^ transfer, and Mg^2+^ coordination. The moderate frustration reported by the glycine and switchable R-R_M_ H-bond residue positions may reflect the flexibility needed for allosteric communication, as speculated (Parra and Ferreiro, 2025).
(3) The aspartyl phosphorylation mediated by a conserved site comprised of an aspartate triad and metal coordination is the universal feature of the RR superfamily, with protein-water H-bonds implicated in the changes triggered by CheY_es_ phosphorylation (Introduction). The present study measures the coupling between key bond transitions and the role of water in Mg^2+^ coordination, in contrast to previous network models (Wang et al., 2020; Wheatley et al., 2020). It shows that protein water H-bonds are a ubiquitous feature of the phosphorylation site over all representatives. The increased REC domain network connectivity of the phosphorylation site is, in addition, driven by the D57-PO_3_^−^ couplings to K109 and T87. It is replicated in two other RRs. We suggest that this connectivity could be generic for the RR superfamily.

(4) The CheY_es_ MD trajectories record undocumented rare events that provide additional details on communication via the hydrophobic core. The T87 and Y106 form water-H-bonds upon phosphorylation. The W58 reorientation has long been a diagnostic of activation, but the simulations show the W58 switching transitions between residue partners responsible for it's reorientation. This evidence implies that the REC core breathes to enable water internalization and bulky sidechain motions. The breathing may, in addition, modulate the hydrophobic relay involved in the regulation of D57 phosphorylation/dephosphorylation kinetics (Foster et al., 2021; Hamid et al., 2026).
(5) The CheY_es_ networks provided additional support for Y-T coupling, but also revealed multiple pathways for information transfer from the target binding surface to the phosphorylation site. Y-T coupling did not occur in the two other RR networks we studied. We could associate the difference between the CheY_es_ and CheY4_vc_ RR models with experimental evidence. The transcriptional regulator 5TQJ network was different from either network. Y106 was a significant network node, but connected to D57 in the network models of the basal but not phosphorylated 5TQJ configuration, inconsistent with CheY_es_ Y-T coupling.

### 3.2 Limitations of the study

It remains to be shown that the relation between residue energy frustration and switchable bond states documented for the RR REC domain is a generic feature of protein families that mediate signal transduction. The functional significance of the difference in the dynamics documented between RRs also needs to be studied with in silico mutagenesis screens supplemented with the construction and experimental characterization of additional mutations to extend current knowledge, particularly for RR transcriptional regulation. The high-throughput capacity of the Frustraevo and Bridge2 tools we have used should facilitate these screens.

We did not study allosteric communication in the hydrophobic core defined by the Frustraevo readout. There is experimental evidence for such a role (Foster et al., 2021). It could contribute to the difference between the overall C^α^ and the more restricted H-bond centrality profiles (Fig. 5A). Inspection of the PC1, PC2 components suggests the β-sheet twists, a generic motion mode (Fenwick et al., 2014), but its amplitude is small relative to the loop and α-helix motions, consistent with its minimal frustration.

An understanding of water dynamics has facilitated drug design as documented by a growing number of studies (Samways et al., 2021; Spyrakis et al., 2017). Drug development against RRs is an attractive proposition since this superfamily does not have mammalian homologs. It has been difficult since available metrics have had limited success in discriminating between subfamilies ((Gao et al., 2007), this study (Fig. 2C,D). It should be facilitated by the tools developed in this study that, as far as we are aware, is the first comprehensive examination of protein-water H-bond dynamics in bacterial RRs.

## 4 Methods

### 4.1 Databases & webservers

Sequences and structures were downloaded from (InterPro, UniProt) and (PDB, Foldseek), respectively. The REC Pfam PF00072 (InterPro IPR001789) database has over 2 million entries. The seed MSA sequences and associated Foldseek models (*n* = 52) were combined with PDB structures (<2 Å resolution (*n* = 138)) for an initial dataset that was subsequently filtered based on sequence and fold similarity. Online servers for additional MSA and manual curation, plus 3D-structure alignment, modification, and in silico mutational analysis of single residue positions are listed in Table 2 (Webservers / Databases). Software installed in-house for sequence analysis, structural dynamic analysis and visualization, statistics, and figure preparation is listed under Table 2 (Tools).

**Table 2:** Webservers/Databases/Tools. Available as editable text file. Prepromali was first used by (Pandini et al., 2015), but was not named.

	Name	Article	Link/Version
	Vienna-PTM	Margreitter et al., 2013	http://vienna-ptm.univie.ac.at
	Muscle	Edgar.,2004	https://www.ebi.ac.uk/jdispatcher/msa/muscle
Webservers	MAFFT	Nakamura et al., 2018	https://www.ebi.ac.uk/jdispatcher/msa/muscle
	DALI	Holm., 2022	http://ekhidna2.biocenter.helsinki.fi/dali/
	FrustraEvo	Parra et al., 2024	https://frustraevo.qb.fcen.uba.ar/
			
	PDB	Burley et al., 2025	https://www.recb.org/
Databases	UniProt	The UniProt Consortium	https://www.uniprot.org/
	CATH	Waman et al., 2024	https://www.cathdb.info/
	Gromacs 2021.4	Pallet al., 2020	https://doi.org/10.5281/zenodo.5636522
	Gromos54a8	Margreitter et al., 2017	https://www.gromos.net/
	VMD1.9.4a57	Humphrey et al.,1996	https://www.ks.uiuc.edu/Research/vmd/
	Bridge2	Siemers & Bondar.,2021	https://github.com/maltesie/bridge2
	Mutlnf	McClendon et al., 2009	https://simtk.org/projects/mutinf/
	OHM	Wang_et.al., 2020	https;//www.ohm.dokhlab.org
	Glocks 0.91b	Talavera., 2007	https://github.com/kbaseapps/kb_gblocks
	Prepromali	This Study	
Tools	CD-Hit 4.8.1	Fu et al., 2012	https://github.com/weizhongli/cdhit/
	HMMER3.3.2	Fin et al., 2011	http://hmmer.org
	AlphaFold2	Jumper et al., 2021	https://hpc.nih.gov/apps/alphafold2.html
	MATLAB 2021b		https://www.mathworks.com/products/matlab.html
	Python 3.7.13		https://www.python.org/
	Cytoscape 3.10.2	Shannon., 2023	https://cytoscape.org/
	Pymol2.4.1		https://www.pymol.org/
	Chem3D Pro12.0		https://revvit-ysignals.com/products/research/chemdraw

### 4.2 Structural Variation in the RR superfamily

The Pfam PF00072 response regulator receiver (REC) domain seed sequences (*n* = 52) MSA was used as the template. The sequences for RR crystal structures from the Protein Data Bank (PDB) with better than 2.0 Å resolution were added to the PF00072 seed MSA with the MAFFT-Add utility (Nakamura et al., 2018). The crystal structure database was a heterogeneous mix of free and metal bound, as well as basal and activated state structures. The latter were functional mimics modified by PO_3_^−^ analogs or residue substitutions. Foldseek AlphaFold models (Jumper et al., 2021) of the PF00072 sequences were combined with the PDB structures to create a 3D structure dataset to match the combined MSA. The MSA was preprocessed by trimming to the template CheY sequence with a custom code (Pandini et al., 2015), termed Prepromali (Preprocessor for Multiple Alignment), to eliminate residue positions absent from the PDB query sequence, 2CHE.pdb, the basal state template for the RR domain. The trimmed sequences were clustered based on 1D-sequence similarity. We selected CD-Hit (Fu et al., 2012), among several options (Pavlopoulos et al., 2023), based on previous experience with it on datasets of similar size (Khan, 2022). CD-Hit is based on the principle of short word filtering that circumvents assumptions used for sequence alignment algorithms to identify and cluster similar sequences (Li and Godzik, 2006). The cluster representatives were analyzed for local frustration with Frustraevo (Freiberger et al., 2023; Parra et al., 2024) and filtered by fold similarity with DALI (Holm, 2022).

The Frustraevo server was built on the Frustrameter algorithm (Ferreiro et al., 2007; Parra et al., 2016) described previously (Hamid et al., 2022). Frustraevo extends the central concept of local frustration as assessed by a decoy distribution of in silico mutants to map the evolution of the local frustration at the single residue level in protein families. The single residue frustration information content, FrustIC. is

(1) $$\mathrm{FrustIC}=H_{\max}-H_{\mathrm{observed}}$$

The observed conformational entropy, $H_{\mathrm{observed}}$, for this residue position in the MSA of family members will be

(2) $$H_{\mathrm{observed}}=-\sum_{i=1}^{M}P_{i}\log_{2}P_{i}$$

where $P_{i}$ is the probability for the i^th^ (M =3) state (minimal, neutral, maximal) defined relative to the decoy distribution obtained by substitution of all residue types in this position with the contact and solvent accessibility values of the surrounding residues fixed (Freiberger et al., 2023). The maximum entropy$,H_{\max}$ was computed using the non-uniform background probabilities determined earlier (Ferreiro et al., 2007), with $\sum_{i=1}^{M}P_{i}$ normalized to 1.

We decomposed $H_{\mathrm{observed}}\;$to obtain the combined FrustIC (${\mathrm{FrustIC}}_{\mathrm{Comb}}$) that subtracts out the minimal state probabilities for comparison between the combined frustration and residue H-bond states.

(3) $${\mathrm{FrustIC}}_{\mathrm{Comb}}=\left({\mathrm{FrustIC}}_{\mathrm{Max}}\right)-\left({\mathrm{FrustIC}}_{\mathrm{Min}}+{\mathrm{FrustIC}}_{\mathrm{Neu}}\right)$$

The DALI server uses intramolecular distance matrices to compare 3D-structures (Holm and Sander, 1993; Holm and Sander, 1995). It can detect remote homologs, assisted with hidden Markov models (Karplus et al., 1997), in experimental and AlphaFold predicted protein databases (Holm et al., 2023). The correspondence between structures were reported as eigenvector heatmaps and correspondence plots. The 59 CD-Hit cluster representatives were resolved into 10 groups and 1 outlier in the PC1-PC2 space using the K-means module available in the Python *Scikit-Learn (sklearn)* machine learning library. The outlier was removed.

K-means partitions the N structures represented by 2D coordinates in DALI eigenvector $\left(\mathrm{eigen}1,\mathrm{eigen}2\right)\;$space. The optimal number of clusters, $gC_{\mathrm{opt}}$ is obtained by minimization of the cost function *J*(g*C*), the aggregate intra-cluster sum of squares.

(4) $$J\left(\mathrm{gC}\right)=\sum_{i=0}^{N}\min_{x\in C}\left({\left\|m_{i}-\mu_{i}\right\|}^{2}\right)$$

where $\mu_{i}$ is the $\left(\mathrm{eigen}1,\mathrm{eigen}2\right)\;$centroid of the grouped cluster, $gC_{i}$, and $m_{i}$ is the centroid of cluster $C_{i}$ within $gC_{i}$. Biological functions of the 10 representative structures output from DALI were compared with the CATH database (Sillitoe et al., 2021). The central CATH module performs structural classification of determined and predicted protein domains into homologous superfamilies (Bordin et al., 2024; Bordin et al., 2023). The superfamilies are further classified into functional families (“FunFams”) (Das et al., 2015; Rojano et al., 2022) based, in part, on the gene ontology database (Attrill et al., 2019). The FunFam functional predictions for the 10 DALI representatives aligned with function as established by experiments (Foster et al., 2021), but there was redundancy with any one sequence assigned to multiple FunFam families. This result reflected the fact that the RR fold is not a sensitive readout of its function (Gao et al., 2007). We merged two pairs of the DALI representatives with very similar FunFam profiles. Principal component analysis (PCA) PC1-PC2 plots (*gmx anaeig*) of the component members identified the average C^α^ backbone fold for each merged group. In one case, a member that overlapped with the average fold (0,0) coordinate in PC1-PC2 space was selected as representative of one merged set. In the other case, an AlphaFold model of the average C^α^ fold was constructed by assigning the predominant residue in the group MSA to residue positions. The final 8 structures were used for molecular dynamics (MD) simulations and network analysis. The selection of the combined RR database down to the final representative structures, and the two-stage analysis of its energetics and dynamics are schematized (Supplementary Fig. S5A). The analyses of the dynamics are detailed below.

### 4.3 Molecular Dynamics

#### 4.3.1 Computer operating systems

The long (1 μs) simulations of the 2CHE and 1FQW_PO3-_ template structures run on the Biowulf supercomputer with GROMACS 2024-gcc11.3 were allocated 2 compute nodes (4xNVIDIA, A100 GPUS (80 GB VRAM, 6912 cores, 432 tensor cores)). The long simulations of the 3CHY template (0.74 ± 0.13 μs) were done on the NUST supercomputing facility (2 x Intel 6-core Xeon E5–2630 V2 2.60GHz, 12 cores, 64GB RAM, with 4 Nvidia-Tesla K10-GPU option). The remaining short (20 ns, 40 ns) simulations were performed within a GROMACS 2021.4 installation on Ubuntu 22.04.3 on LUMS BIRL servers (4 Intel Xeon E7–8880 v3 4 × 18-cores, 1024GB RAM).

#### 4.3.2 Parameterization

The BeF_3_^−^ was removed and the D57 residue modified to dianionic D57 aspartyl phosphate in the 1FQW template structure using the Vienna PTM server (Margreitter et al., 2017) to obtain phosphorylated CheY (1FQW_PO3-_). The database representative structures were modified by the addition of Mg^2+^ to create the Mg^2+^-bound basal state, and the D57 phosphorylated, as for the template (1FQW_PO3-_), to create Mg^2+^-bound phosphorylated states. The GROMACS operations for the simulations and the subsequent analysis of the network dynamics are outlined below**.**

Water was parameterized after file conversion (*gmx pdb2gmx*) as the SPC 3-point model. The model has been studied as a function of temperature and long-range interaction cutoff (Mark and Nilsson, 2002). SPC is one of multiple water models, none of which completely reproduce the properties of water (Kadaoluwa Pathirannahalage et al., 2021). The proteins were solvated in a cubic water box with 1 nm separation between the protein and box boundaries (*gmx solvate*). The box was adjusted to neutral pH (7.0), adjusted to 150 mM ionic strength by addition of Na^+^ and Cl^−^ ions (*gmx genion*), and checked for molar water density (*gmx energy*).

#### 4.3.3 Preproduction

The system was equilibrated as instructed in the GROMACS tutorial (*https://tutorials.gromacs.org/docs/md-intro-tutorial.html*). In brief, energy minimization using the steepest descent algorithm was followed by NPT (isothermal-isobaric ensemble) (100 ps) and NVT (canonical ensemble) equilibration (100 ps). Temperature control was set at 300 K and 0.1 ps coupling constant with the Berendsen algorithm. Isotropic pressure was set at 1 bar with the Parrinello-Rahman barostat. Nonbonded interactions had a 1.0 nm Verlet cutoff, with the long-range interactions approximated by the particle-mesh Ewald (PME) method. We checked that the frame-by-frame root C^α^ root-mean-square deviations (RMSDs) reached a stationary value. This value, averaged over all energy minimization runs, was 0.03 ± 0.00 nm.

*Production.* The production runs (*gmx mdrun*) used structure files created (*gmx grompp*) from the same parameter and topology files as the preproduction runs to process the output from the pressure equilibration. The 1 μs simulations used a 2 fs integration step. These simulations analyzed C^α^ backbone dynamics. The 20 ns simulations used a 1 fs integration step, to capture water dynamics as used by the Bridge developers (Karathanou et al., 2020). Simulations were performed in triplicate for each structural state (3 templates plus 8 × 2 database representatives). Both 1 μs and 20 ns simulations were performed for the template structures (2CHE, 1FQW_PO3-_) to assess the finite sample size error. The proteins were centered, then rotationally and translationally-aligned in the structure (*gmx editconf*) and trajectory (*gmx trjconv*) files. Index files (*gmx make_ndx*) edited these output files for concatenation (*gmx trjcat*), removal of water or other components as needed.

### 4.4 Construction of the protein-water H-bond network

A complete identification of H-bonds and water positions based on visual inspection of protein structures is challenging so automated methods have been developed for this purpose. Here, we extended the use of Bridge2 (Siemers and Bondar, 2021) introduced in our initial study (Hamid et al., 2022) to analyze MD trajectories (Supplementary Fig. S5B). The use of k-d trees is central to the computational power of Bridge2 that renders it two orders of magnitude faster than other options (Siemers et al., 2019) such as GROMACS *gmx hbond*. In common with other programs, Bridge2 uses a ≤ 3.5 Å bond distance and 60° angle to search for H-bonds. The frame-by-frame occupancy of the identified H-bonds is the primary output of the search. The algorithm also computes secondary metrics from this primary output - network measures, long-range contiguity, water-wires and H-bond motifs. However, the display of these metrics by the Bridge2 GUI is expensive in computer memory for large trajectory files. Therefore, we downloaded the raw occupancy data and wrote our own code to analyze and visualize metrics of interest. We validated our code by matching the network centrality with Bridge2 using structure files alone. The scripts were validated by comparison against the network output by the Bridge2 GUI in the former case, and the test dataset supplied in the latter case.

We then used the Bridge2 occupancy data to first characterize the water-mediated coordination of the Mg^2+^ and its modulation by D57_PO3-_. We computed water diffusion coefficients in the absence of protein (free mobility) as another benchmark of the SPC water model, then compared the position-dependent mobilities of the waters at the Mg^2+^.D57_PO3-_ binding pocket and other locations reported in the 2CHE and 1FQW crystals against the mobility of free water, as well as the waters added to solvate the protein in the simulation box.

Second, we determined the protein-water H-bond network. In protein network models, the residues form nodes and the interactions between them are edges. The readout of the protein-water H-bond network architecture by the Bridge2 algorithm is the betweenness centrality (Siemers and Bondar, 2021). Importantly, the Bridge2 networks are physical networks where the edges are set by steric constraints (≤3.5 Å, 60°). The residue node centrality reflects the mean occupancies and residence times of its H-bonds formed with water and other residues.

### 4.5 The analysis of dynamic networks

For dynamic networks that are not subject to strict steric constraints, the correlations were measured as the mutual information between dynamic couplings – between residue sidechain rotations (*gmx gangle*), bond distances (*gmx distance*), backbone dynamics (*gmx chi*). The master equation for the normalized mutual information is

(5) $$\mathrm{nMI}\left(X,Y\right)=\left(\frac{\mathrm{MI}\left(X,Y\right)-\varepsilon \left(X,Y\right)}{H\left(X,Y\right)}\right)$$

where X and Y are the relevant residue columns. $\mathrm{MI}\left(X,Y\right)$ is the mutual information, $H\left(X,Y\right)$ is the joint entropy and $\varepsilon \left(X,Y\right)$ is the finite size error.

The mutual information (*MI*) is given by:

$$\mathrm{MI}\left(X,Y\right)=\sum_{x,y}p\left(x,y\right)\log\left(\frac{p\left(x,y\right)}{p\left(x\right)p\left(y\right)}\right)$$

The joint entropy (H) is defined as:

$$H\left(X,Y\right)=-\sum_{x,y}p\left(x,y\right)l{\mathrm{og}}_{2}p\left(x,y\right)$$

The *sklearn* library was used to compute the dynamic couplings between residue sidechain rotation angles and bond distances.

The eigenvector centrality of the C^α^ backbone of the Mg^2+^-free apo-CheY (3CHY.pdb) was characterized earlier based on a structural alphabet that reported secondary structure fluctuations (Wheatley et al., 2020). Here, we used the mutual information (MutInf) between the torsional angles as the network metric for C^α^ backbone dynamics (McClendon et al., 2012), The $\mathrm{MI}\left(X\left(\varphi_{1}\right),Y\left(\varphi_{2}\right)\right)$. The correlations between residue torsional angles (ϕ, Ψ) were computed as the difference between the conformational self-entropies and the joint entropy.

(6) $$H\left(X,Y\right)=\sum_{i}^{n}\int_{0}^{2\pi }p\left(\varphi \right)\ln p\left(\varphi \right)\mathrm{d\varphi}-\sum_{i}^{n}\sum_{j\neq i}^{n}\int_{0}^{2\pi }\int_{0}^{2\pi }p\left(\varphi_{1},\varphi_{2}\right)\ln\frac{p\left(\varphi_{1,}\varphi_{2}\right)}{p\left(\varphi_{1}\right)p\left(\varphi_{2}\right)}\;d\varphi_{1}d\varphi_{2}+\ldots -\ldots$$

where the (…)’s denote higher-order joint entropy terms

PCA (*gmx anaeig*) for isolation of the principal components and cluster analysis (*gmx cluster*) for determination of conformational heterogeneity complemented the network analysis of the C^α^ backbone in the MD trajectories.

The $\left(X,Y\right)$ residue states for computation of the dynamic network associated with H-bond transitions were alphanumeric strings that represented distinct H-bond states, similar to the structural alphabets for secondary structure states. By analogy

(7) $$\epsilon \left(X,Y\right)=\frac{B_{X,Y}-B_{X}-B_{Y}+1}{2N}$$

where N is the sample size, $B_{X},B_{Y}$ are the number of distinct residues X and Y alphanumeric states and $B_{X,Y}$is their joint probability (see (Carmona et al., 2025)). To give a qualitative illustration, consider the coupling between a R-R_S_ residue R1 with a R-R/W residue R2 that bonds alternately with H_2_O and one residue (not R1). The R1 H-bond will be either OFF or ON, while R2 will have 3 states (OFF, H_2_O ON, residue ON). The nMI will be zero if the 3 R2 states exhibit no preference for whether or not R1 forms a H-bond, and have a maximum value 1, if one R2 state occurs only when the R1 H-bond is ON.

The “Identify allosteric pathways and critical residues” option in the OHM was used for construction of the perturbation propagation networks. The aspartate triad (D12, D13, D57) was defined as the active site. The Y106, K91 pair was defined as the allosteric site. Seven snapshots at 10 ns intervals from the concatenated 60 ns trajectories were sampled. The allosteric coupling intensity (ACI) scores were averaged, sorted and normalized by the aggregate of all reported couplings. The top ACI scores (> (μ + 3σ)) are reported.

We used betweenness centrality rather than eigenvector centrality for a direct comparison of the fold conformational landscape with the H-bond dynamics. Both centrality measures were defined earlier by (Wheatley et al., 2020) and (Hamid et al., 2022), respectively. In brief, eigenvector centrality, taken as the eigenvector of the adjacency matrix, measures the connectedness of a node. The betweenness centrality of a node is proportional to the fraction of the shortest paths between other nodes that include it (Rodrigues, 2019). Construction of the covariance matrix (*gmx covar*) preceded the determination of the dynamic couplings and centrality measures. These metrics measured the modulation of the long-range allosteric network by the solvent water at single residue resolution, which, in concert with the physical H-bond network, compared the allosteric network architecture in the RR superfamily. The betweenness centrality, rather than eigenvector centrality, was used for a direct comparison of the fold conformational landscape with the H-bond dynamics. Construction of the covariance matrix (*gmx covar*) preceded the determination of the dynamic couplings and centrality measures.

### 4.6 Quantitative and statistical analysis

The significance evaluation for the DALI distance matrices and the FrustrameterR decoy distributions is detailed in the citations (Holm and Sander, 1993; Parra et al., 2016).

Linear and non-linear best fits to the data, with standard errors, were computed in MATLAB. Pearson correlation and *t*-tests were used to compare distributions from replicates within and between different water populations (Fig. 4). Differences (Δ_bas, pho_) between the basal (bas) and phosphorylated (pho) CheY_es_ MutInf centrality for successive 3-residue fragments were evaluated against the square root of the combined variance (σ ^1/2) (Fig.S1B).

For significance, modulus (Δ_bas, pho_) > (σ ^1/2), where

σ=σ1^2+σ2^2+σ3^2/3bas+σ1^2+σ2^2+σ3^2/3pho)

The background probabilities of the H-bond groups as observed over the basal and phosphorylated alignments (Fig. 5b) were obtained to assess the significance of the state frequencies observed for relevant residue positions and the correlation between FrustIC and H-bond group propensity. The Y-T coupling for basal and phosphorylated CheY_es_ was reported by the mutual information matrix (*n* = 6^2) normalized as the nMI to correct for finite-size error (Fig. 1). Networks for each replicate trajectory were constructed to assess the robustness of network features (Fig. 6) as reported by the centrality variations (Fig. S3a). The significance of the long-range nMI couplings associated with the H-bond state transitions (Fig. 8) was assessed by a two-step procedure. Decoy distributions generated with custom Python scripts utilized NumPy and Pandas libraries to randomly shuffle residue H-bond states across 1000 iterations to generate a null distribution. The initial coupling matrix (*n* = 122^2) was filtered to retain only couplings that exceeded the (mean + 2σ) value of the null distribution (n 〈1000). The couplings that exceeded the (mean + 2σ) of the filtered distribution were used for network construction, repeated for all three replicates as well as the cumulative trajectory (Fig. S3b).

## Figure preparation

Atomic structure images and movies were prepared in PyMol. Chemical bonds were shown with ChemDraw-3D. Cytoscape was used for the 2D representations of the H-bond networks.

## CRediT authorship contribution statement

**Maham Hamid:** Writing – original draft, Visualization, Validation, Software, Methodology, Investigation, Formal analysis, Data curation. **Ishrat Jabeen:** Writing – review & editing, Supervision, Software, Resources, Methodology, Formal analysis. **Safee Ullah Chaudhary:** Writing – review & editing, Software, Resources, Project administration, Methodology, Funding acquisition, Formal analysis. **Shahid Khan:** Writing – review & editing, Writing – original draft, Validation, Supervision, Project administration, Methodology, Investigation, Funding acquisition, Formal analysis, Conceptualization.

## Declaration of competing interest

The authors declare that they have no known competing financial interests or personal relationships that could have appeared to influence the work reported in this paper.

## Data Availability

Data and scripts have been deposited on GitHub and Figshare. GitHubHydrogenBond_Analysis (Original data)
FigshareThe hydrogen bonded network in bacterial response regulators (Original data)

## Appendix A Supplementary data

Supplementary material: The protein-water H-bond network for bacterial response regulator REC domain activation.
