# Supplementary material for: The protein-water H-bond network for bacterial response regulator REC domain activation
Source: J Struct Biol X. 2026 Sep 19;14:100164. doi: 10.1016/j.yjsbx.2026.100164 (PMC13636085; doi:10.1016/j.yjsbx.2026.100164)
Supplement: Supplementary file 1 [file mmc1.pdf]

Revision-2

SUPPLEMENTARY MATERIAL

The protein-water H-bond network for bacterial response regulator REC domain activation

FIGURES

Figure S1. CheYes C^{α} conformational landscapes and networks.
Figure S2. Structural determinants of water mobility
Figure S3. Network node and edge variations across replicates
Figure S4. Long-range dynamic couplings.
Figure S5. Methodology for RR superfamily assembly, filtration, and analysis

VIDEOS

Video S1. 2CHE hydrogen-bonded network.
Video S2. $1FQW_{PO3-}$ hydrogen-bonded network.

PSE Files

Fig.5C. FrustIC
Fig.5C. H-bond group
Fig.S1B. $1FQW_{PO3-}$ Centrality
Fig.S1C. $1FQW_{PO3-}$ OHM network

REPOSITORIES

The custom scripts and MutInf validation have been deposited on GitHub (*https://github.com/BIRL/HydrogenBond_Analysis*).
The videos, structure files, trajectories and analysis datasets have been deposited on Figshare. They can be accessed via a private link (*https://figshare.com/s/aa4f086a07b14a047883*).
The Figshare repository will be made public once the article has been accepted for publication.

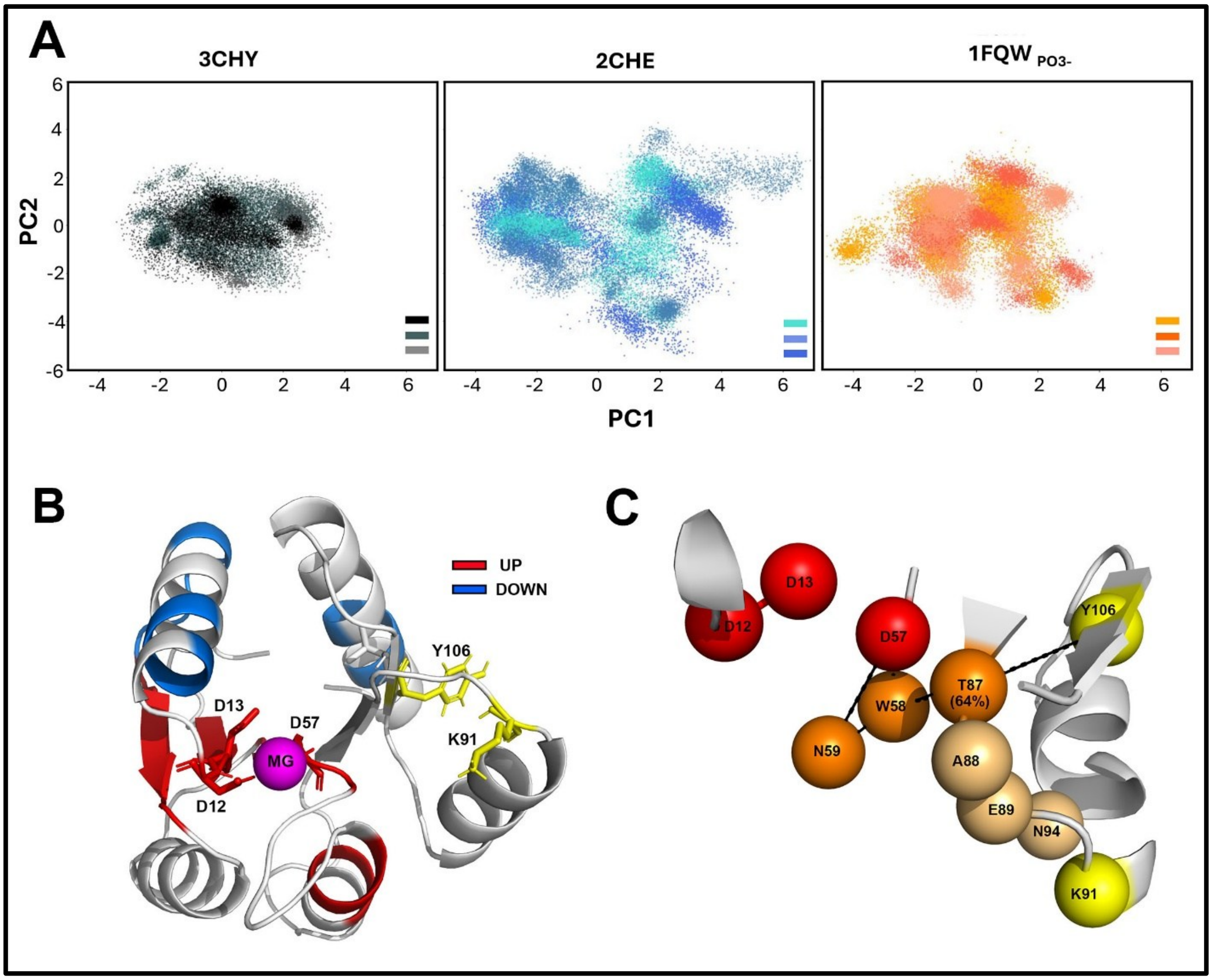

Figure S1: A. Principal component analysis of CheY$_{es}$ conformational landscapes. *Variations between replicate (n = 3) trajectories obtained for the three different CheY forms – apo (3CHY), basal, Mg^{2+}-bound (2CHE)) and phosphorylated, Mg^{2+}-bound (1FQW$_{PO3}$-). 1μs 2CHE and 1FQW$_{PO3}$- replicates. 3CHY (0.6, 0.63, 1μs) replicates. Different shades (horizontal bars) represent different replicates. Comparison of the 3CHY and 2CHE panels suggests that, after correction for trajectory length, bound Mg^{2+} expands the accessible conformational landscape. Comparison of the 2CHE and 1FQW$_{PO4}$ panels suggests that phosphorylation selects, rather than increase, the basal CheY substates in the landscape.* ***B. Mutual information network centrality****. 3x20ns replicates, 3-residue fragments. Upregulated ((red)) and downregulated ((blue)) segments were obtained from the comparison of the difference between the centrality profiles (Fig. 5A) against their combined variance (****Methods****).* ***C. Contact network****. The top pathways (>mean + 3σ) between the active (D12, D13, D57(red)) and allosteric site (Y106, K91(yellow)) reported by the OHM server[1] for the phosphorylated configuration. Y106 <- T87 <- W58, N59 <- D57 (ACI=0.026 (orange)). K91 <- N94 <- E89 <- A88 <- T87 <- D57 (ACI=0.057 (light-brown))., The corresponding pathways (Y106 <- D57 (ACI=0.011), K91<- D57 (ACI=0.029)) are weaker and do not involve T87 for the basal configuration. See PSE files for B, C.*

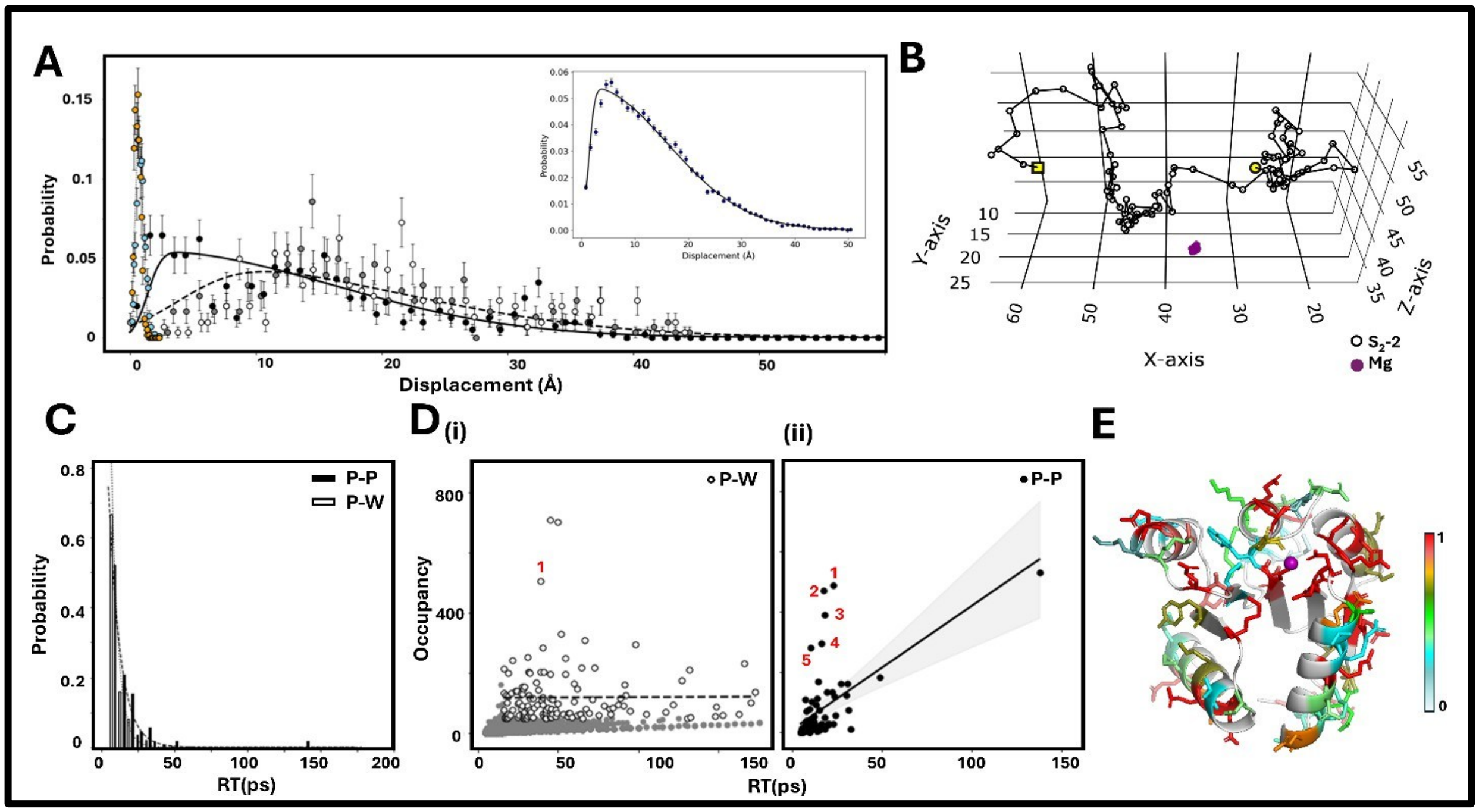

Figure S2: Structural determinants of water mobility. A, B. Mg^{2+} coordination/phosphorylation site. A. *Mobility of crystal waters plotted as the distribution of the distance (mean +/- se) from the starting coordinates in successive frames over the 500ps period (3 replicates). Mg^{2+} coordination: 1st shell (2CHE (cyan, n=3), $1FQW_{PO4}$ (orange, n=2) Fit (dotted line) $\left(0.3\sqrt{2\pi}\right)exp\left(-\left(\frac{x-0.756)^2}{2.0.3^2}\right)$. Peak distance = 0.76Å. Radial distances after energy minimization: 1st Shell (1.96 ± 0.009 Å), 2nd shell (29.1$\pm$1.14Å). 2^{nd} shell waters reset to crystal water positions (4.5$\pm$0.7Å) for production runs. Skewed Gaussian fits (dashed line $2 * \phi\ (x) * \Phi(4.4 * x)$; solid line $2 * \phi\ (x) * \Phi(16.0 * x)$, where x =distance, ϕ = probability density function, Φ = cumulative distribution function.* ***Inset*** *shows fit to "other" crystal waters. Compare with the solid line 2nd shell water fit.* ***B****. Example 500ps displacement trajectory of a 2nd shell water (yellow symbols mark start (circle) and end (square)).* ***C-E. H-bonds. C.*** *Residence time (RT) distributions for P-P and P-W H-bonds (3 replicates, 4 ns duration, 5ps intervals). Mg^{2+} water shells excluded.* ***D.*** *The correlation between H-bond occupancy (Occ) and residence time (RT).* ***(i)*** *P-W bonds. H-bonds that remain (open symbols) or are eliminated (grey symbols) by the occupancy filter (0.05). Fit (dashed line) Occ=0.02(RT)+118.1. Non-crystal water trapped at 2nd water shell location (asterisk). (ii) P-P bonds. Fit (solid line with 95% confidence region shaded gray) Occ = 4.09(RT)+9.7.* ***P-W.*** *D12-HOH_{3948}(1)). HOH_{3948} acts as a 2nd shell water for a substantial fraction of one 4 ns trajectory, with 6.03$\pm$0.06Å distance from the Mg^{2+} ion over the final 3.1 ns.* ***P-P*** *(R18-E35(1), D41-K45(2), T112-T115(3), M17-K109(4), R22-E35(5)).* ***E****. Water contact map after 1^{st} persistence filter (> 3 frames). Residues that form P-W bonds (stick sidechains) are color-coded based on their P-W occupancy (Bar). Acidic (D, E) residues had the highest occupancy (0.95$\pm$0.01 (n=18)), followed by basic (K, R) residues (0.54$\pm$0.6 (n=15)). T87 occupancy was notably higher (0.83) than other threonine residues (0.43$\pm$0.11(n=5)). Two aromatic residues (W58 (0.20), Y106 (0.61)) also formed P-W bonds. Methionine has the lowest occupancies (0.10$\pm$0.04 (n=6)). Other polar residues (N, S, Q) made up the remaining population (0.56$\pm$0.05 (n=15)). There was no correlation with the SASA (P_{CC} = -0.16). 4 ns 2CHE trajectory.*

A

2CHE	P0A2D52	4H6O	Q6BBK6_VIBCL	5TQJ	B2JEF9_PARP8
Residue	Uniprot Nbr	Residue	Uniprot Nbr	Residue	Uniprot Nbr
		M	1		
		N	2		
M	1	E	3	M	1
A	2	G	4	G	2
D	3	N	5	T	3
K	4	T	6	N	4
E	5	M	7	K	5
L	6	A	8	P	6
K	7	K	9	F	7
F	8	V	10	V	8
L	9	L	11	V	9
V	10	A	12	V	10
V	11	V	13	V	11
D	12	D	14	D	12
D	13	D	15	D	13
F	14	S	16	D	14
S	15	I	17	A	15
T	16	S	18	S	16
M	17	I	19	V	17
R	18	R	20	G	18
R	19	Q	21	R	19
I	20	M	22	A	20
V	21	V	23	I	21
R	22	S	24	R	22
N	23	H	25	R	23
L	24	T	26	L	24
L	25	L	27	L	25
K	26	Q	28	R	26
E	27	D	29	S	27
L	28	A	30	V	28
G	29	G	31	G	29
F	30	Y	32	I	30
N	31				
N	32	E	33	A	31
V	33	V	34	A	32
E	34	E	35	D	33
E	35	T	36	T	34
A	36	A	37	Y	35
E	37	A	38	T	36
D	38	D	39	S	37
G	39	G	40	G	38
V	40	R	41	D	39
D	41	E	42	E	40
A	42	A	43	F	41
L	43	L	44	L	42
N	44	A	45	D	43
K	45	K	46	V	44
L	46	A	47	L	45
Q	47	Q	48	S	46
A	48	K	49	A	47
G	49	A	50	T	48
				P	49
				S	50
				Y	51
G	50	R	51	R	52
F(Y)	51	F	52	P	53
G	52	D	53	D	54
F	53	V	54	C	55
I(V)	54	I	55	V	56
I	55	I	56	I	57
S	56	S	57	L	58
D	57	D	58	D	59
W	58	V	59	V	60
N	59	N	60	Q	61
M	60	M	61	M	62
P	61	P	62	P	63
N	62	V	63	G	64
M	63	M	64	S	65
D	64	T	65	N	66
G	65	G	66	G	67
L	66	F	67	I	68
E	67	E	68	E	69
L	68	F	69	V	70
L	69	V	70	Q	71
K	70	K	71	R	72
T	71	A	72	R	73
I	72	V	73	L	74
R	73	R	74	A	75
A	74	M	75	G	76
D	75	Q	76	G	77
S(Δ)	76	S	77	-	-
A	77	Q	78	-	-
M	78	Y	79	-	-
S	79	K	80	-	-
A	80	F	81	A	78
L	81	T	82	V	79
P	82	P	83	P	80
V	83	I	84	V	81
L	84	L	85	I	82
M	85	M	86	F	83
V	86	L	87	I	84
T	87	T	88	T	85
A	88	T	89	A	86
E	89	E	90	H	87
A	90	T	91	D	88
K	91	S	92	D	89
K	92	P	93	A	90
E	93	E	94	G	91
N	94	K	95	V	92
I	95	K	96	R	93
I	96	Q	97	E	94
A	97	E	98	T	95
A	98	G	99	A	96
A	99	K	100	L	97
Q	100	A	101	A	98
A	101	V	102	A	99
G	102	G	103	G	100
A	103	A	104	A	101
S	104	T	105	R	102
G	105	G	106	A	103
Y	106	W	107	Y	104
V	107	L	108	L	105
V	108	V	109	R	106
K	109	K	110	K	107
P	110	P	111	P	108
F	111	F	112	F	109
T	112	N	113	N	110
A	113	P	114	D	111
A	114	E	115	V	112
T	115	T	116	L	113
L	116	L	117	F	114
E	117	L	118	I	115
E	118	K	119	R	116
K	119	T	120	T	117
L	120	L	121	V	118
N	121	Q	122	C	119
K	122	R	123	A	120
I	123	V	124	V	121
F	124	L	125	L	122
E	125			G	123
K	126			I	124
L	127			A	125
G	128			A	126
M	129			P	127
				L	128

B

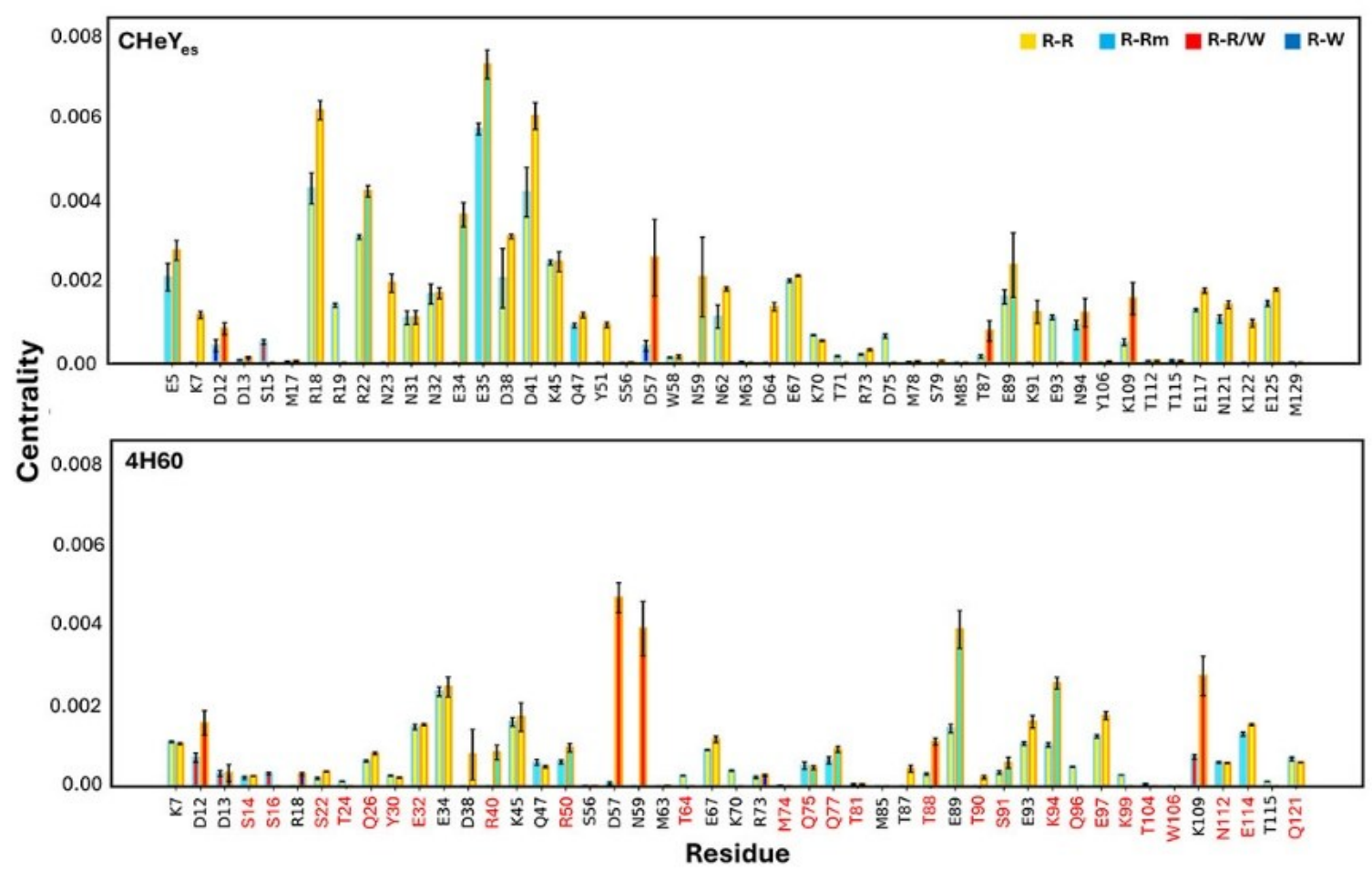

C

(i)

Residue 1	Residue 2	NMI_rep1	NMI_rep2	NMI_rep3	Mean_NMI
ASP-13	ASP-57	0.401115594	0.046694258	0.004019843	0.150609898
ASN-31	ASN-32	0.120534716	0.00422582	0.148451917	0.091070818
ASP-75	MET-78	0.085432218	0.124638044	0.032989419	0.081019894
ASP-57	GLU-93	0.052693461	0.166665557		0.073119672
ASP-57	ASN-94	0.07987905	0.083982271		0.05462044
ASP-13	GLU-93	0.055126643	0.096328902		0.050485182
ASP-13	TRP-58	0.145579967			0.048526656
ASP-13	ASN-94	0.087526757	0.05442177		0.047316176
ASP-13	MET-85	0.133383658	0.001746446	0.003162451	0.046097518
ASP-57	TRP-58	0.137874423			0.045958141
ASP-57	MET-85	0.126264342	0.003004713		0.043089685
TRP-58	ASN-59	0.117613115			0.039204372
ASN-59	MET-85	0.112179955			0.037393318
MET-17	SER-56	0.093516235		0.015678892	0.036398376
MET-63	MET-85	0.060967592	0.03376762	0.001284697	0.032006637
THR-71	MET-85	0.060967592	0.03376762	0.001284697	0.032006637
ASP-38	SER-56	0.022521303	0.042660997	0.021555893	0.028912731
SER-56	ASN-62	0.022521303	0.042660997	0.021555893	0.028912731
SER-56	TRP-58	0.086025245			0.028675082
ASP-13	THR-87	0.080645988	0.001423807	0.000654078	0.027574624
SER-56	MET-85	0.079788818			0.026596273
MET-85	ASN-94	0.063322255	0.009530951	0.006238233	0.026363813

CheY$_{es}$

Residue -1	Residue-22	NMI-rep1	NMI-rep2	NMI-rep3	Mean-NMI
ASN-94	TYR-106	0.086828098	0.114675473	0.143510389	0.115004653
ASN-31	ASN-32	0.013019051		0.138312315	0.050443789
ASP-38	ASN-94	0.028471435	0.002163852	0.091774319	0.040803202
ASN-62	ASN-94	0.028471435	0.002163852	0.091774319	0.040803202
TRP-58	MET-85	0.000536238	0.112210634	0.003549475	0.038765449
ASP-38	ASP-57	0.004283874	0.007735315	0.100554776	0.037524655
ASP-57	ASN-62	0.004283874	0.007735315	0.100554776	0.037524655
ASP-57	ASN-94	0.020657399	0.01519357	0.066746136	0.034199035
LYS-7	TYR-51	0.01478119	0.049197963	0.037663026	0.033880726
SER-56	ASP-57	0.026302511		0.072620202	0.032974237
MET-85	TYR-106	0.033049731	0.056865854	0.005114607	0.031676731
ASP-12	ASP-57	0.087111298	0.00217788	0.001683085	0.030324088
ASP-38	GLU-89	0.035618542	0.007017775	0.044320024	0.028985447
ASN-62	GLU-89	0.035618542	0.007017775	0.044320024	0.028985447
TYR-51	ASN-94	0.025653465	0.028094983	0.028929809	0.027559419
THR-87	TYR-106	0.029840475	0.041526326	0.010241935	0.027202912
ASP-57	TYR-106	0.019969291	0.016042908	0.043679106	0.026563769
ASP-38	TYR-51	0.01253717		0.067027379	0.026521516
TYR-51	ASN-62	0.01253717		0.067027379	0.026521516
ASN-94	LYS-109	0.038059154	0.031565105	0.007596625	0.025740295
ASP-12	MET-17	0.057825196	0.007532021	0.010903227	0.025420148
TYR-51	GLU-89	0.030293445	0.008241962	0.03749943	0.025344946
TRP-58	TYR-106	0.006287508	0.061880847	0.007607912	0.025258756
GLU-34	ASN-94	0.021633888	0.029298967	0.023973848	0.024968901
SER-56	THR-87	0.048613553		0.022191229	0.023601594
THR-87	ASN-94	0.018752513	0.040619291	0.011280986	0.02355093
SER-56	ASP-64	0.000472207	0.05475717	0.011573273	0.02226755
ASP-38	TYR-106	0.003487384	0.023086854	0.03955696	0.022043733
ASN-62	TYR-106	0.003487384	0.023086854	0.03955696	0.022043733
ARG-73	THR-87	0.033028488	0.032249545		0.021759344
SER-79	THR-87	0.033028488	0.032249545		0.021759344
SER-56	GLU-89	0.043492864	0.002505738	0.018357993	0.021452199
GLU-34	GLU-89	0.020988169	0.014178629	0.02753151	0.020899436
GLU-5	TYR-106	0.05234808	0.003949096	0.00560313	0.020633435
SER-56	ASN-94	0.038388218	0.002380092	0.019465981	0.020078097

(ii)

Residue 1	Residue 2	NMI_rep1	NMI_rep2	NMI_rep3	Mean_NMI
ASP-57	LYS-109	0.072088591	0.424048598	0.076836195	0.190991461
ASP-12	ASP-57	0.165995844	0.084685697	0.204203981	0.151628507
SER-16	ASP-57	0.122548319	0.181478951	0.018639837	0.107555702
ASP-12	LYS-109	0.033826349	0.094964589	0.183874114	0.104221684
ASP-12	THR-104		0.200828194	0.099550638	0.100126278
SER-14	LYS-109	0.032703673	0.001075886	0.223608404	0.085795988
ASP-12	SER-16	0.160341904	0.016064278	0.072964945	0.083123709
ASP-12	THR-64	0.014087497	0.180706738	0.016058071	0.070284102
ASP-12	GLU-67	0.014087497	0.180706738	0.016058071	0.070284102
SER-16	THR-104		0.172843069	0.026105716	0.066316262
SER-14	ASP-57	0.120437543	0.001190145	0.073979688	0.065202459
THR-24	THR-104		0.001609382	0.174688159	0.058765847
SER-16	LYS-109	0.004909229	0.138857605	0.026551119	0.056772651
ASP-12	THR-81	0.148652104	0.013186542	0.006769063	0.056202569
ASP-12	ARG-73	0.141743393	0.001828708	0.001822506	0.048464869
ASP-57	ARG-73	0.125163611	0.003080668	0.004356519	0.044200266
SER-56	GLN-75	0.006001836	0.008357811	0.116372178	0.043577275
ASP-57	THR-81	0.107258845	0.0104041	0.009787461	0.042483469
ASP-13	SER-16	0.028447424	0.074494796	0.020279267	0.041073829
SER-16	THR-64	0.009370471	0.101891573	0.011943285	0.041068443
SER-16	GLU-67	0.009370471	0.101891573	0.011943285	0.041068443
ASP-57	THR-104		0.034482974	0.081808484	0.038763819

4H60

Residue-1	Residue-2	NMI-rep1	NMI-rep2	NMI-rep3	Mean-NMI
ASN-59	THR-88	0.207318775	0.199866244	0.215087733	0.207424251
THR-87	THR-88		0.018186916	0.509229333	0.175805416
ARG-18	THR-87	0.009602716		0.38978105	0.133127922
ASP-57	THR-88			0.375677143	0.125225714
ASP-57	LYS-109		0.031651571	0.340975993	0.124209188
ARG-18	SER-56	0.00668898		0.365790785	0.124159255
ASP-57	ASN-59	0.025480123	0.31094791	0.030239087	0.122222373
THR-87	LYS-109			0.359776142	0.119925381
SER-56	THR-87	0.001047618	0.003409313	0.332703337	0.112386756
SER-56	THR-88	0.032413673	0.010592154	0.285917053	0.10964096
SER-56	ASP-57	0.046931368	0.014246415	0.265579566	0.108919116
THR-88	LYS-109		0.006910492	0.309861676	0.105590723
ARG-18	THR-88	0.003468341	0.001584705	0.308515492	0.104522846
ASP-57	GLU-89	0.044370601	0.192155422	0.06819725	0.101574424
ARG-18	ASP-57	0.02798979		0.271535439	0.099841743
ARG-18	LYS-109			0.255573383	0.085191128
SER-14	THR-88	0.080208	0.17017473	0.004057304	0.084813345
ASP-12	ASP-57	0.074500869	0.167989719	0.009882085	0.084124224
SER-56	LYS-109		0.008073558	0.239496273	0.082523277
ASP-12	THR-88	0.07993608	0.144915957	0.007466767	0.077439601
SER-14	ASP-57	0.074659502	0.15157431	0.005153034	0.077128949
ASP-12	ASN-59	0.058814773	0.16243956	0.007967234	0.076407189
SER-56	ASN-59	0.037044031	0.017233554	0.172771229	0.075682938
ASN-59	LYS-109		0.042115616	0.179970168	0.074028595
ASN-59	THR-87	0.001442897	0.00452878	0.206614244	0.070861973
ASP-12	GLU-89	0.041831639	0.162217446	0.005859015	0.069969367
ARG-18	ASN-59	0.01712397		0.182794478	0.066639483
THR-88	GLU-89	0.007366274	0.131339805	0.061153574	0.066619885
ASP-57	LYS-94	0.018476829	0.152311843	0.019719106	0.063502593
THR-87	LYS-109			0.359776142	0.119925381

Figure S3. Network node and edge variations across replicates. A. *Residue index key. Residue positions were numbered according to the UniProt Sequences. Bracketed entries show E. coli (1FQW) entries that differ from S. typhimurium (2CHE). The 4H6O residues were renumbered to the matched 2CHE positions in the Fig.5B MSA alignment (cyan, query sequence (4H6O)). Red letters identify 4H6O residues not conserved with 2CHE. The UniProt numbering for 5TJQ was retained. Residues absent in the crystal structure (orange) or excluded from the MSA (yellow) are shown.* ***B.*** *Phosphorylation dependent effects on network nodes. Paired bar histograms (+/- standard error across 3 replicates) show the difference between the basal ((Left; cyan outlines) and phosphorylated (right: orange outlines) configurations for $CheY_{es}$ and 4H6O. The bar fills denote H-bond group.* ***C.****. Tables (Basal (Left) / Phosphorylated (Right) of the significant* ***(i)*** *$CheY_{es}$ and* ***(ii)*** *4H6O nMI couplings, across 3 replicates (Rep1, Rep2, Rep3) ranked by nMI score. Replicate scores > cutoff threshold (green). Replicate scores < 20% cutoff threshold (cyan). Highlighted $CheY_{es}$ nodes (D57 (maroon), N94 (yellow), Y106 (orange)), 4H6O nodes (D57 (maroon), T87, T88 (brown), K109 (yellow)).*

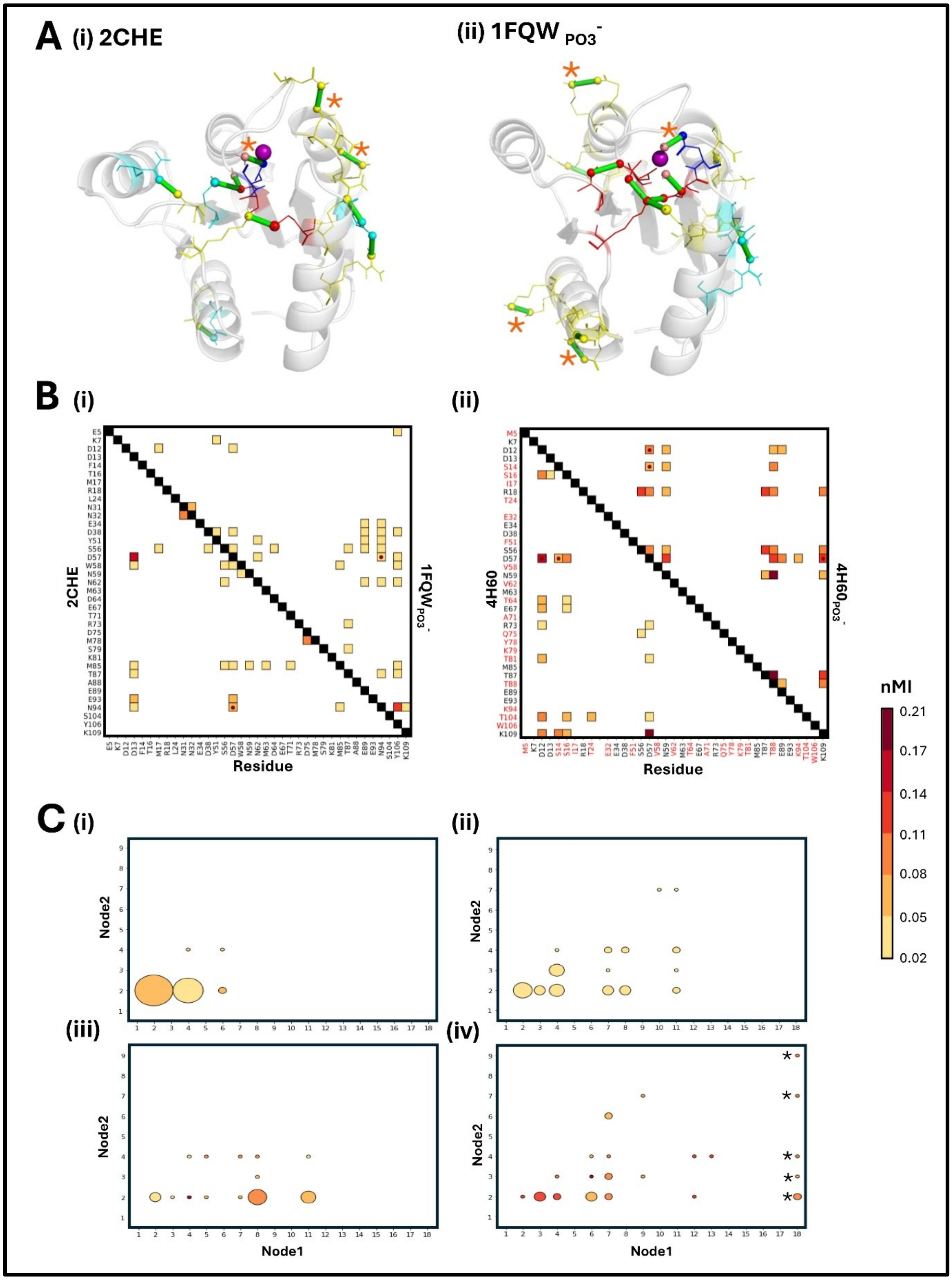

***Figure S4. A. The coupling between H-bond and backbone fluctuations. (i). 2CHE (Video S1 still). (ii). 1FQW$_{PO4}$ (Video S2** still). Sidechains (line representations) of the central H-bond (green bars) residues identified by Bridge2 with the bonding atoms (spheres) color-coded by group. Mg^{2+}*

(magenta). Water (salmon). Residue groups ((iii) R-R_M (cyan); (iv) R-R/W (red)) form multiple H-bonds simultaneously or alternately with other residues. Residue groups ((i) R-W (blue); (iii) R-R_S (yellow)) have single H-bond partners. H-bond associations/dissociations are tied to the peripheral α–helix motions. ***B. Difference matrices of the nMI couplings between basal (bottom left) and phosphorylated (top right) forms. (i) $CheY_{es}$ (ii) $CheY_{vc}$.*** *Significant couplings, identified by comparison against a decoy distribution, are shown. The number and coupling strength increase upon phosphorylation. For* ***$CheY_{es}$,*** *Y106 transitions from the uncoupled state in the basal configuration to multiple couplings, notably with T87, D57, N59, and N94; while T87 is coupled to Y51, R73, S79, and N94 in addition to Y106. For* ***$CheY_{vc}$*** *T87, T88, uncoupled in the basal configuration, form multiple couplings, notably with D12, R18, D57, N59, and K109. Red font marks $CheY_{vc}$ residue substitutions relative to $CheY_{es}$. $CheY_{vc}$ residue positions have been renumbered based on the MSA to correspond to $CheY_{es}$ positions. Symbol color indicates nMI value (Bar). Red dots mark couplings common to both basal and phosphorylated configurations.* ***C. State multiplicity for the dynamic couplings. (i)*** *2CHE.* ***(ii)*** *$1FQW_{PO4}$.* ***(iii)*** *4H6O.* ***(iv)*** *$4H6O_{PO4}$. State multiplicity denotes the number of distinct H-bond states formed in the analyzed MD simulation trajectory. The number will be 2^n for a residue node with n atoms that form H-bonds (H-$bond_{MAX}$ = 2) each with a single partner, or greater if the atoms bond with multiple partners. For coupled residue nodes, the number for the node with the higher count is plotted on the x-axis, and that for the lower count node on the y-axis. Symbol size represents the number of distinct couplings with the same, and color represents the mean nMI value (Bar). Single H-bond (n=2) couplings are most common. Phosphorylation increases the state multiplicity, a possible consequence of the increase in the number and coupling strength. In addition, the aspartyl phosphate can form more H-bonds than aspartate. The increase is particularly evident in the $4H6O_{PO4}$ D57 aspartyl phosphate mediated couplings (asterisks (*)). 3x20 ns trajectories per configuration.*

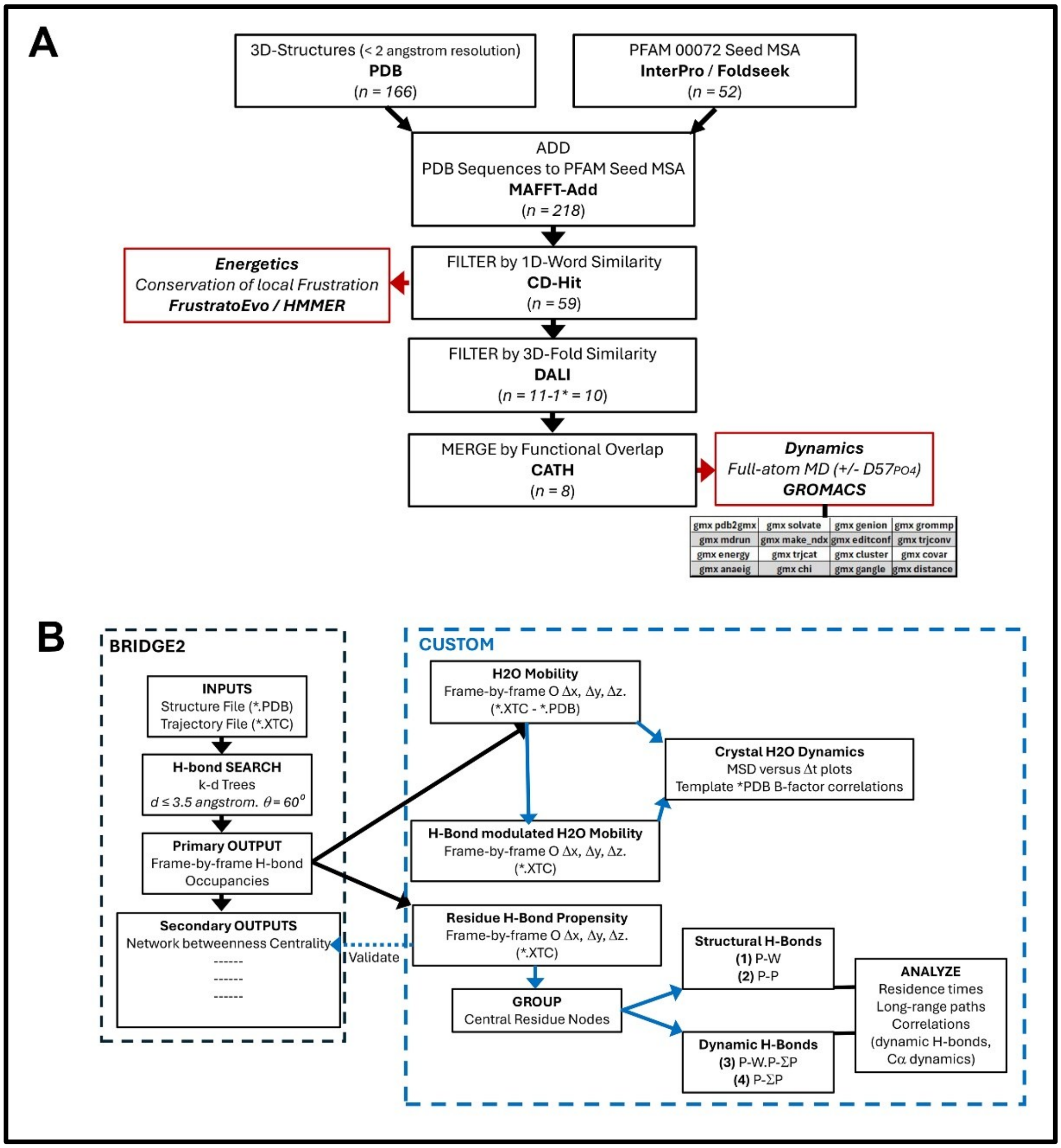

Figure S5: A. Methodology for RR superfamily construction, filtration, and analysis. *The workflow for the construction of the composite 1D sequence and associated 3D structure database, and the 4 stages for selection of structures for analysis is shown. For simulations of the dynamics, each structure selection was simulated with and without the phosphoaspartyl modification in the presence of* Mg^{2+}*. See text for the GROMACS operators used for the MD simulations and analysis.* ***B. The Bridge2 H-bond protein-water network.*** *The workflow for the metrics extracted from the Bridge2 output.*

1 Wang, J. *et al.* Mapping allosteric communications within individual proteins. *Nat Commun* **11**, 3862, doi:10.1038/s41467-020-17618-2 (2020).
